# Supplementary material for: The Acrasis kona genome and developmental transcriptomes reveal deep origins of eukaryotic multicellular pathways
Source: Nat Commun. 2024 Nov 25;15:10197. doi: 10.1038/s41467-024-54029-z (PMC11589745; doi:10.1038/s41467-024-54029-z)
Supplement: Supplementary file 1 — Supplementary Information [file 41467_2024_54029_MOESM1_ESM.pdf]

The Acrasis kona genome and developmental transcriptomes reveal deep origins of eukaryotic multicellular pathways

Sheikh et al.

Supplemental Tables and Figures

Table S1. Genome statistics for *Acrasis kona* and various eukaryotes
Table S2. The *Acrasis kona* genome assembly
Table S3. *Acrasis kona* genome completeness as measured by A) CEGMA and B) BUSCO
Table S4. Classes of repeat sequences and their frequencies
Table S5. Genomes used for orthologous clustering of *A. kona* protein families
Table S6. Summary statistics for KEGG mapping and comparison of metabolic pathways
Table S7. Developmental signaling pathways in aggrgegating *A. kona*
Table S8. Documented instances of aggregative multicellularity in eukaryotes

Figure S1. Metabolic capacity of Acrasis kona and Naegleria gruberi
Figure S2. Variation by time point in protein function profile for *Dictyostelium discoideum* homologs of *Acrasis kona* aggregation proteins
Figure S3. An *Acrasis kona* aggregation-induced phosphoinositide phospholipase C acquired from Fungi
Figure S4. Phylogenetic support for *Acrasis kona* aggregation proteins showing evidence of horizontal transfer
Figure S5. Sequence conservation and developmental expression of the *Acrasis kona* exosome
Figure S6. The *Acrasis kona* kinetochore and life cycle-stage specific expression patterns
Figure S7. The central cell divison regulatory proteins CDC20 and CDH1 of *Acrasis kona* are both CDC20s
Figure S8. External signaling proteins predicted among novel *Acrasis kona* Aggup accessions
Figure S9. The Acrasis kona genome annotation pipeline

Table S1. Genome statistics for *Acrasis kona* and various eukaryotes.

Species	Genome size (mB)	%GC	Protein-coding loci	% coding	% genes with introns	Introns per gene	Median intron length
Acrasis kona	44	38	15868	45.5	48	1.1	40
Naegleria gruberi	41	57	15727	57.8	36	0.7	60
Homo sapiens	2851	41	23328	1.2	83	7.8	20383
Neurospora crassa	40	54	10107	36.4	80	1.7	72
Dictyostelium discoideum	34	22	13574	62.2	68	1.3	236
Arabidopsis thaliana	140.1	36	26541	23.7	80	4.4	55
Thalassiosira pseudonana	34.5	47	11242	ND	ND	1.4	ND
Saccharomyces cerevisiae	12	38	6039	73	ND	1	141

Table S2. The *Acrasis kona* genome assembly including A) general genome statistics, and B) summary statistics for gene annotation based on evidence, *ab initio* and manual curation (Supplementary Fig. S9).

2A. Genome assembly statistics	
Number of contigs	2039
Genome size	44.02 Mb
GC content	38%
N50	41.9Kb
Coverage	55X
Number of genes	15868
Number of exons	32530
Total gene length	20 x 10^6
Total exon length	19 x 10^6
Mean gene length	1269
Mean exon length	591
Mean intron length	42
Mean exons per mRNA	2.1
Mean mRNA per gene	1
% of genome coding	45.5

2B. Genome anmnotation statistics	Ab-intio	Evidence	Ab-intio + evidence + manual
Gene number	13952	9574	15868
3' UTR number	4550	8279	8624
5' UTR number	5042	9413	8751
Exon number	30281	29587	32530
Total gene length	17 x 10^6	11 x 10^6	20 x 10^6
Total exon length	16 x 10^6	13 x 10^6	19 x 10^6
Mean gene length	1254	1216	1269
Mean exon length	553	416	591
Mean intron length	46	50	42
Mean exons per mRNA	2.17	2.56	2.1
Mean mRNA per gene	1	1,2	1
% of genome coding	39	26	45.5

Table S3. ***Acrasis kona*** **genome completeness as measured by A) CEGMA and B) BUSCO.**

**A) Genome completeness as measured by CEGMA (Para et al. 2007)**					
	**Acrasis Protein number**	**Acrasis percentage completeness**	**Acrasis Total**	**Acrasis Average**	**Acrasis Percentage Orthologs**
**Complete**	221	89.11	308	1.39	28.51%
**Group 1**	56	84.85	72	1.29	21.43%
**Group 2**	50	89.29	65	1.3	26,00%
**Group 3**	54	88.52	77	1.43	31.48%
**Group 4**	61	93.85	94	1.54	34.43%
**Partial**	230	92.74	337	1.47	31.74%
**Group 1**	60	90.91	84	1.4	28.33%
**Group 2**	52	92.86	74	1.42	28.85%
**Group 3**	57	93.44	84	1.47	35.09%
**Group 4**	61	93.85	95	1.56	34.43%

**B) Genome completeness as measured by BUSCO (Simão et al. 2015)**					
**Lineage: Eukaryota**	***Acrasis kona predicted genes***	***Naegleria gruberi predicted genes***		***Acrasis kona genome assembly***	***Naegleria gruberi genome assembly***
**Complete BUSCOs (C)**	282	257		209	257
**Complete and single-copy BUSCOs (S)**	249	243		186	253
**Complete and duplicated BUSCOs (D)**	33	14		23	4
**Fragmented BUSCOs (F)**	7	15		23	7
**Missing BUSCOs (M)**	14	31		71	39
**Total BUSCO groups searched**	303	303		303	303

References:

Parra G, Bradnam K, Korf I. CEGMA: a pipeline to accurately annotate core genes in eukaryotic genomes. Bioinformatics 23(9):1061-7 (2007).

Simão FA, Waterhouse RM, Ioannidis P, Kriventseva EV, Zdobnov EM. BUSCO: assessing genome assembly and annotation completeness with single-copy orthologs. Bioinformatics 31(19):3210-2 (2015).

Table S4. Classes of repeat sequences and their frequencies in the *Acrasis kona* genome.

Repeat type	Number	size (kb)	mean size (kb)	% genome
AF321458_1	1	0.28	285	0
AF104021_1	1	0.32	318	0
AF478691_1	1	0.37	368	0
NP_009811.1	1	0.41	411	0
AAD09018.1	2	0.44	220.5	0
BAB40830.1	2	0.45	225	0
AF227253_1	2	0.47	234	0
S33633	1	0.49	495	0
CAD12894.1	2	0.59	297	0
AF104027_1	2	0.61	307.5	0
AF104025_1	2	1.27	633	0
CAA31790.1	6	1.51	251.5	0
AAA46576.1	8	2.23	278.25	0
A40831	10	6.13	612.9	0.01
AAG13367.1	9	6.43	714.22	0.01
DNA/TcMar-Ant1	32	15.56	486.12	0.03
DNA/Kolobok-T2	26	21.89	841.96	0.05
DNA/Crypton	20	22.32	1116.1	0.05
LTR/ERV1	57	27.7	486	0.06
DNA/TcMar-ISRm11	42	30.39	723.57	0.07
LTR/Ngaro	44	31.75	721.61	0.07
DNA/MuLE-NOF	17	31.82	1871.53	0.07
LINE/L1-Tx1	22	37.26	1693.55	0.08
LINE/R1	26	39.34	1512.88	0.09
LTR/Copia	40	42.17	1054.22	0.09
LTR/Pao	77	47.2	613.01	0.11
DNA/TcMar-IS885	61	48.24	790.77	0.11
DNA/PIF-Harbinger	54	49.61	918.69	0.11
Low_complexity	1030	52.38	50.85	0.12
DNA/Zator	73	57.69	790.25	0.13
LTR	79	61.13	773.84	0.14
LINE/Penelope	48	62.43	1300.58	0.14
DNA/MuLE-F	90	80.2	891.1	0.18
DNA/Sola	200	92.68	463.38	0.21
DNA/PiggyBac	121	93.75	774.83	0.21
LINE/R2-NeSL	86	95.18	1106.78	0.21
LINE	224	127.19	567.8	0.28
DNA/MuLE-MuDR	158	157.77	998.52	0.35
LTR/DIRS	269	180.61	671.42	0.4
DNA/hAT-Ac	371	219.72	592.23	0.49
LINE/L1	334	259.87	778.04	0.58
LINE/I	412	294.43	714.63	0.66
Simple_repeat	5593	296.05	52.93	0.66
LINE/R2	276	330.46	1197.31	0.74
rRNA	470	351.62	748.13	0.79
LTR/Gypsy	474	358.23	755.75	0.8
DNA/CMC-EnSpm	620	484.42	781.32	1.08
Unknown	5321	3387.26	636.58	7.57
total				16.8

Table S5. Genomes used for orthologous clustering of the *Acrasis kona* predicted proteome. Sequences were clustered using OrthoMCL (Li et al. 2003).

Organism	Taxonomy	Assembly version	Availability
Neurospora crassa	Fungi		NCBI Genome
Batrachochytrium dendrobatidis JAM81		v1.9	NCBI Genome
Acanthamoeba castellanii	Amoebozoa	Acastellanii.strNEFF v1	NCBI Genome
Dictyostelium discoideum		dicty_2.7	NCBI Genome
Monosiga brevicollis	Holozoa	V1.0	NCBI Genome
Drosophila melanogaster		Release 6 plus ISO1 MT	NCBI Genome
Thalassiosire pseudonana	Stramenopila	ASM14940v2	NCBI Genome
Phytophthora infestans		ASM14294v1	NCBI Genome
Aureococcus anophagefferens		v1.0	NCBI Genome
Paramecium tetraurelia	Alveolata	ASM16542v1	NCBI Genome
Vitrella brassicaformis		Vbrassicaformis	NCBI Genome
Chlamydomonas reinhardtii	Archaeplastida	v3.0	NCBI Genome
Ostreococcus tauri		v050606	NCBI Genome
Arabidopsis thaliana		TAIR10	NCBI Genome
Naegleria gruberi	Discoba	assembly v1	NCBI Genome
Percolomonas cosmopolitus strain WS		Transcriptome	iMicrobe
Percolomonas cosmopolitus strain AE-1		Transcriptome	iMicrobe
Andalucia godoyi		Transcriptome	Baldauf lab
Seculomonas ecuadoriensis		Transcriptome	Baldauf lab

Reference: Li L, Stoeckert CJ Jr, Roos DS. OrthoMCL: identification of ortholog groups for eukaryotic genomes. Genome Res. 13(9):2178-89 (2003).

Table S6. Summary Statistics for KEGG mapping and comparison of metabolic pathways in *Acrasis kona* and *Naegleria gruberi* (Supplementary Fig. S1). Full detail in Supplementary Data 3.

	KO number	Unique	Metabolic	Regulatory	Secretory biosynthesis
Acrasis kona	2529	453	776	606	168
Naegleria gruberi	2532	456	802	573	169
**Enzyme overlap**	2076	-	647	533	141
**Total**	-	-	931	646	196

Table S7. Developmental signaling pathways in aggregating *Acrasis kona* (Fig. 8). *A. kona* accessions with substantially increased expression during aggregation (Aggup) and other key components with RPKM >100 during aggregation expression are shown schematically in Fig. 8A and in detail in Fig. 8B (external signaling pathways) and below (internal signaling pathways). Details of sequence annotation, supporting evidence and gene expression levels are shown in Supplementary Data 6 and 13.

signaling pathway		protein		*Acrasis kona*			*Ddi AX4*
**pathway**	**components**	**protein name**	**acronym**	**accession**	**RPKM Agg**	**DE Log2 Gro_Agg**	**DE Log2 0_5**
PKB-TOR	TOR	*target of rapamycin TOR*	TOR	*AKO1_011982*	*29.17*	*0.06*	0.69
		target of rapamycin TOR	TOR	*AKO1_011977*	*82.17*	*0.49*	0.69
		CRAC_Rictor	Rictor	AKO1_003073	18.88	2.23	▲ 1.90
		small GTPase Arf-like 3	Arf	AKO1_005926	19.37	1.86	▲ 1.48
		Arf_exchange factor_cytohesin	ArfGEF	AKO1_000009	102.42	0.94	▲ 1.29
		Rac activator	RacGAP	AKO1_011632	37.74	2.49	▲ 1.06
		Rac activator	RacGAP	AKO1_013552	56.92	0.94	▲ 2.09
		Rac activator	RacGAP	AKO1_011221	235.54	0.91	▲ 1.06
		small GTPase Scar_Rac		AKO1_010272	3.16	2.31	0.15
sphingosine		non-lysosomal ceramidase	CDase	AKO1_006065	603.42	1.19	▲ 4.49
		neutral sphingomyelinase	SMase	AKO1_004193	124.56	0.90	0.33
		sphingosine_1P_lyase	"	AKO1_011426	194.55	1.16	0.56
		NAPE-PLD	PLD	AKO1_000039	46.06	1.91	0.81
		Pro-saposin	saponin	AKO1_009907	136.00	1.17	▼ -1.04
		Ras_KRas	Ras family	AKO1_004265	52.27	1.63	▲ 2.18
		Ras_GAP	"	AKO1_001246	179.14	1.07	▲ 1.64
		Ras_GAP	"	AKO1_005517	56.33	1.07	▼ -2.62
		Ras_GEF	"	AKO1_014844	64.87	1.06	▲ 3.30
kinases	MAPK	MAPKK	MAPKK	AKO1_006554	56.60	0.92	▲ 2.14
		MAPK	MAPK	AKO1_012198	50.30	1.12	▲ 2.41
		MAPK	"	AKO1_015653	8.07	2.40	▲ 2.41
		alpha kinase	"	AKO1_010823	99.70	1.26	▲ 3.44
		GalOx_Ketch	"	AKO1_006253	65.09	1.54	▲ 0.92
		GalOx_Ketch	"	AKO1_011531	16.47	1.99	▲ 2.95
	cell	PKc-like, A-kinase	kinase	AKO1_013190	108.28	1.62	▲ 2.56
	cycle-	Ser/Thr kinase, Aurora	"	AKO1_003728	9.58	2.19	-0.19
		Ser/Thr kinase, CdK2_related	"	AKO1_008411	344.03	0.91	▲ 2.56
		Ser/Thr kinase, Nek2/NimA	"	AKO1_015138	180.1	1.08	▼ -1.13
		Ser/Thr kinase, Polo-like	"	AKO1_008166	43.68	2.94	▼ -1.22
		Ser/Thr kinase	"	AKO1_015703	118.46	1.00	▼ -1.29
phospha-	Ser-The	Ser/Thr phosphatase, ankyrin	S/TP'ase	AKO1_014864	101.18	0.90	▲ 1.86
tases		Ser/Thr phosphatase, SAPS	STKc	AKO1_009583	197.64	0.93	▲ 0.97
		Ser/Thr phosphatase, PP2Cc	"	AKO1_014801	38.81	1.72	▼ -1.53
		Ser/Thr phosphatase, STP4_reg1	"	AKO1_014367	1046.96	0.77	0.65
	Tyr	Tyr phophatase, MKP-4	TyrP'ase	AKO1_009846	18.39	1.50	▼ -1.94
		Tyr phophatase, PTPc	"	AKO1_011663	103.7	1.17	-0.22
	target	C6orf120	cytokine	AKO1_000834	470.8	0.97	-

Table S8. Documented instances of aggregative multicellularity in eukaryotes.

supra kingdom	kingdom	phylum	taxa	reference
Amorphea	Holozoa	Filsterea	*Capsaspora owczarzaki**	Sebé-Pedrós et al. 2013<br>Tikhonenkov et al. 2020
	Holomycota	Rotosphaerida	*Fonticula alba*	Brown et al. 2009
	Amoebozoa	Tubulinea	*Copromyxa spp.*	Brown et al. 2011
		Evocea	Dictyostelia	Raper 1985
Diaphoretickes	Stramenopila	Labyrinthulomycetes	*Sorodiplophrys stercorea*	Tice et al. 2016
	Rhizaria	Cercozoa	*Guttulinopsis vulgaris*	Brown et al. 2012b
	Alveolata	Ciliophora	*Sorogena stoianovitchae*	Sugimoto & Endoh 2008
Excavata	Discoba	Heterolobosea	*Acrasis spp.*	Brown et al. 2012a

*Although *Capsaspora owczarzaki* [59] and close relatives [110] can be induced to form aggregates in the lab, there is no evidence yet of this resulting in cellular differentiation, fruiting body formation or sporulation.

References:

Brown MW, Spiegel FW, Silberman JD. Mol Biol Evol 26:2699-2709 (2009).
Brown MW, Silberman JD, Spiegel FW. Protist. 162(2):277-87 (2011).
Brown MW, Silberman JD, Spiegel FW. Eur J Protistol. 48(2): 103-23 (2012a).
Brown MW, Kolisko M, Silberman JD, Roger AJ. Curr Biol. 22(12):1123-7 (2012b).
Raper K. The Dictyostelids. Princeton University Press (1985).
Sebé-Pedrós A, Irimia M, Del Campo J, Parra-Acero H, Russ C, Nusbaum C, Blencowe BJ, RuizTrillo I. Elife. 2:e01287 (2013).
Sugimoto H, Endoh H. J Eukaryot Microbiol. 53:96-102 (2006).
Tice AK, Silberman JD, Walthall AC, Le KN, Spiegel FW, Brown MW. J Eukaryot Microbiol. 63(5):623-8 (2016).
Tikhonenkov DV, Hehenberger E, Esaulov AS, Belyakova OI, Mazei YA, Mylnikov AP, Keeling PJ. BMC Biol. 18(1):39 (2020).

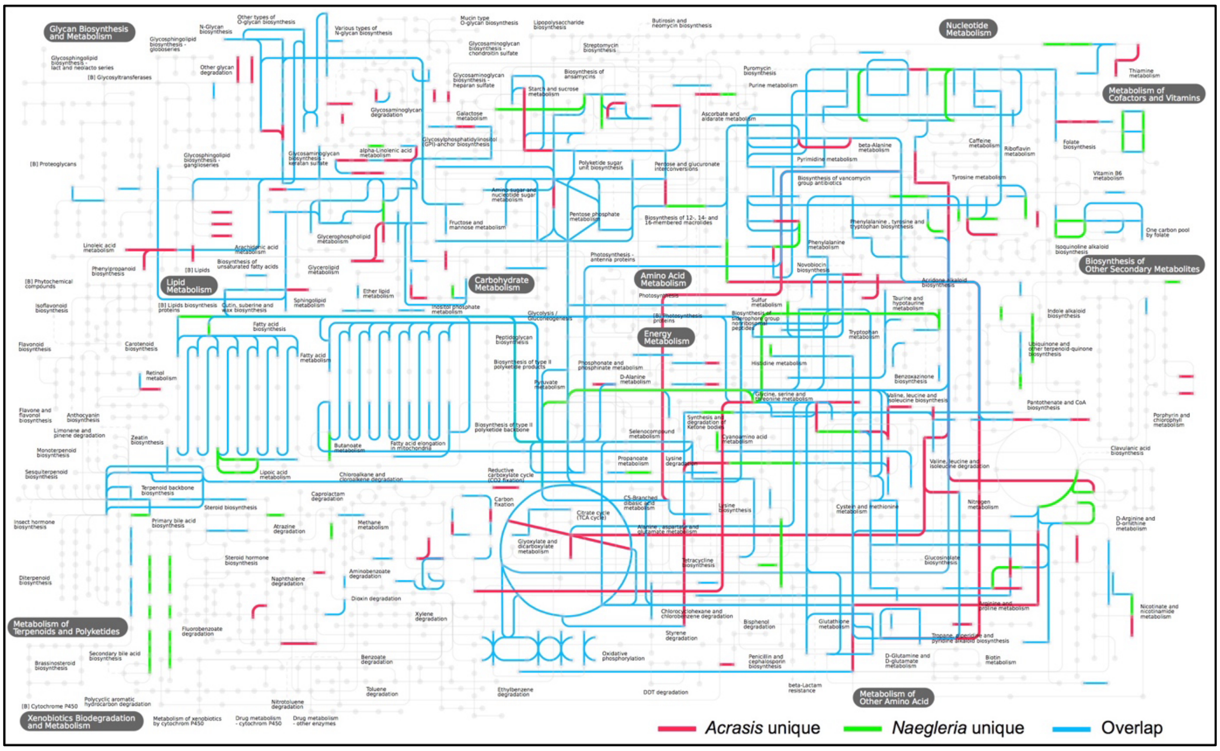

Figure S1. Metabolic capacity of *Acrasis kona* and *Naegleria gruberi*. Unique enzymatic pathways for *A. kona* and *N. gruberi* are shown in red and green, respectively, and the overlap between the two in blue. Pathways were predicted and graphically summarized using iPath (Letunic et al. 2008). A full list of KEGG-predicted *A. kona* metabolic pathways and associated accessions is found in Supplementary Data 3.

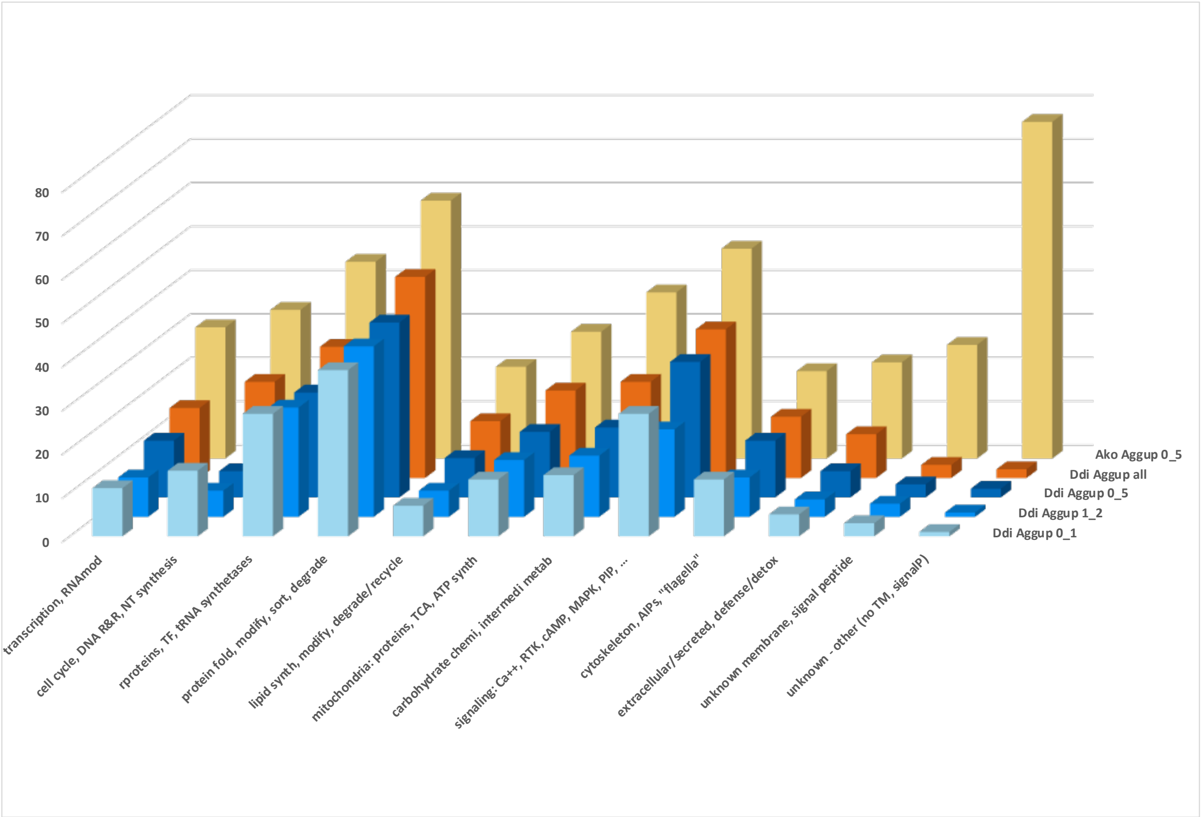

Figure S2. Variation by time point in protein function profile for *Dictyostelium discoideum* homologs of *Acrasis kona* aggregation proteins. *D. discoideum* AX4 (Ddi) proteins homologous to *Acrasis kona* (Ako) Agg_{up} accessions and with substantially increased expression and/or very highly expressed during starvation (as defined in Supplementary Data 6) are clustered into functional categories. Time point comparisons correspond to hours after food depletion: hours 0 vs 1 (0_1), 1 vs 2 (1_2), 0 vs 5 (0_5), all four comparisons combined (all), and all Ako Agg_{up} accessions in growth versus aggregation (0_5, Supplementary Data 6). Bar height (y axis) corresponds to numbers of accessions in major function categories (Supplementary Data 7).

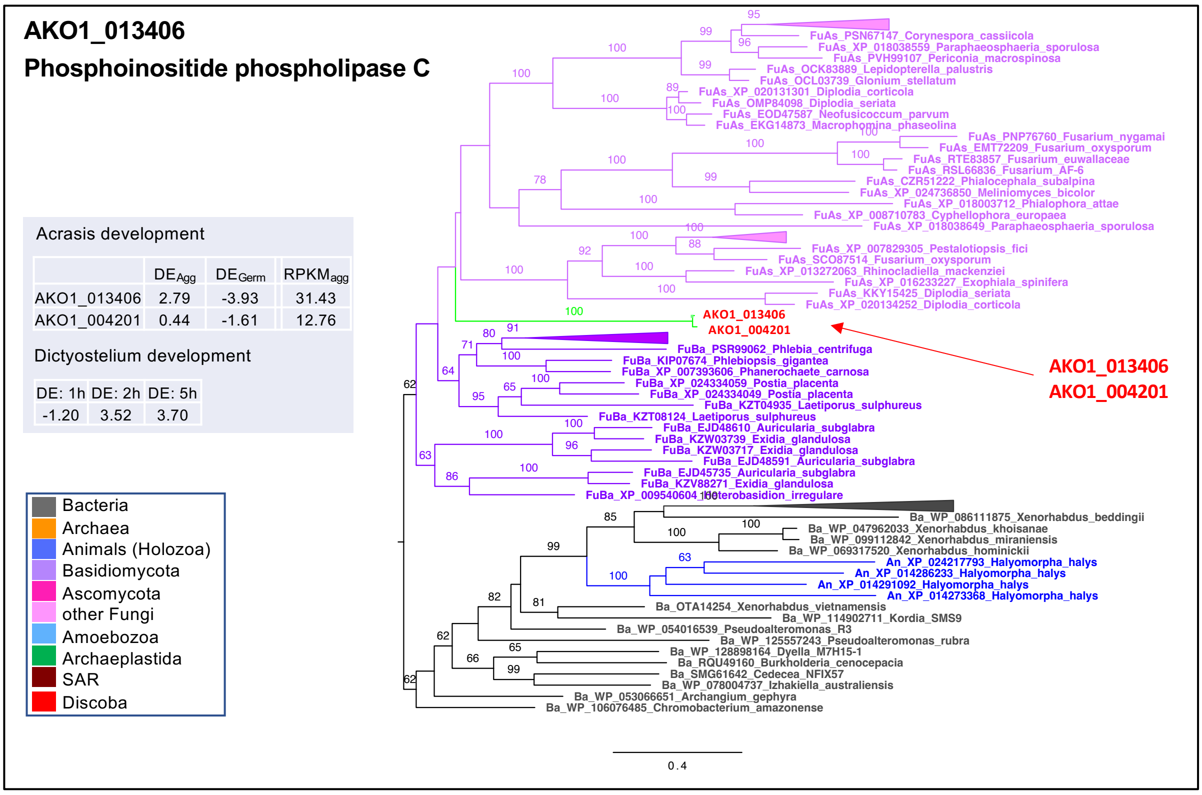

Figure S3. An *Acrasis kona* aggregation-induced phosphoinositide phospholipase C acquired from Fungi. The tree shown was derived from a 415 amino acid alignment using RAxML and the LG+g model [104]. Only bootstrap values over 60% are shown and branch lengths are drawn to scale according to the scale bar at the lower left (substitutions per site). Sequence names are colored according to higher order taxonomy shown in the lower left key. Some large groups of closely related sequences are collapsed to proportional triangles to increase readability of more relevant parts of the trees. The table to the immediate left shows gene expression values for growth vs aggregation (DE_{Agg}), aggregation vs germination. (DE_{Germ}) and numbers of length-corrected reads ($RPKM_{Agg}$) for aggregating cells (Supplementary Data 5 and 7). DE_{Log2} values for Ddi AX4 homologs are given for growth (0 hours) versus 1, 2 and 5 hours starvation (Santhanam et al. 2015).

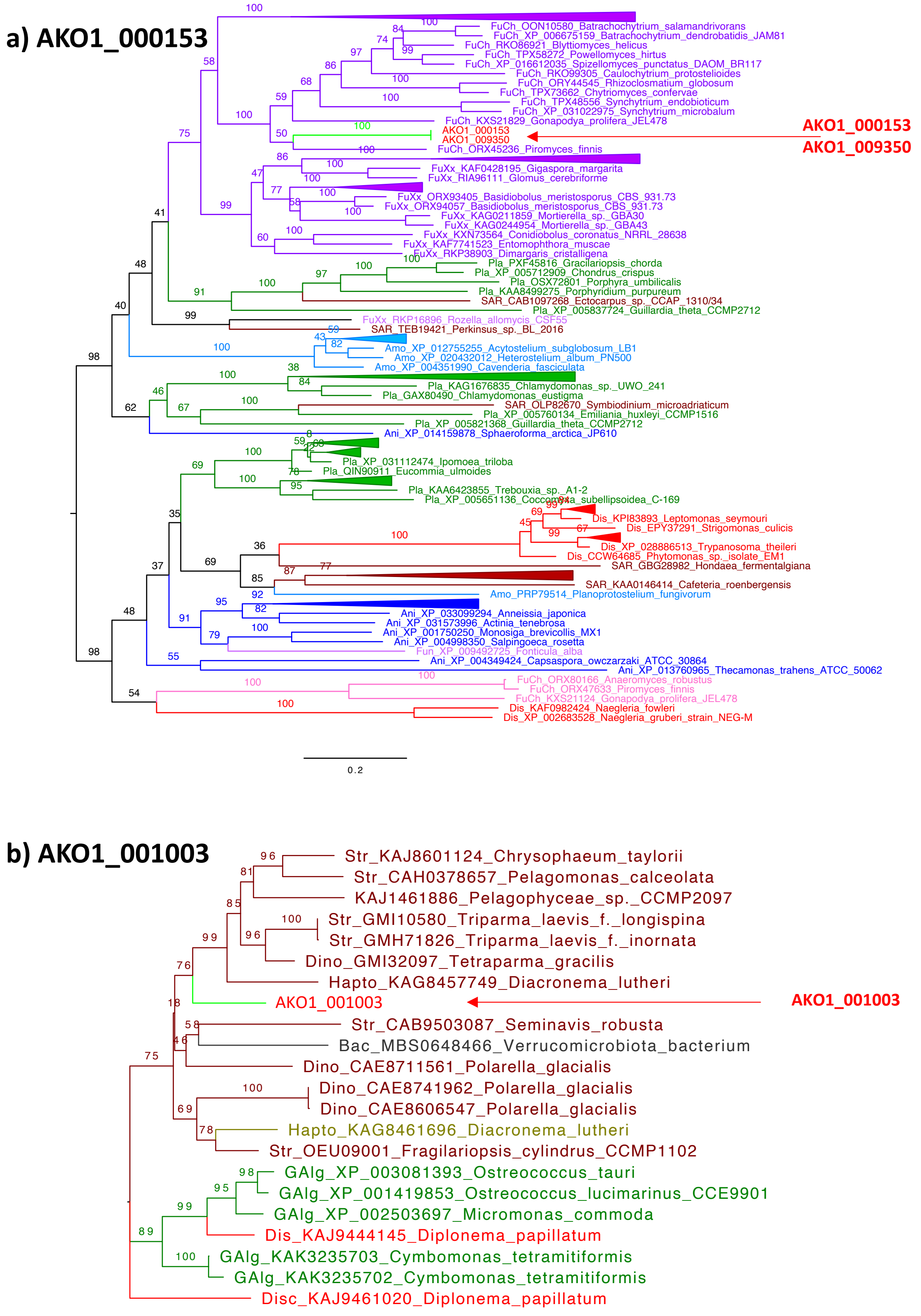

a) AKO1_000153
FuCh_OON10580_Batrachochytrium_salamandrivorans
FuCh_XP_006675159_Batrachochytrium_dendrobatidis_JAM81
FuCh_RKO86921_Blyttiomyces_helicus
FuCh_TPX58272_Powellomyces_hirtus
FuCh_XP_016612035_Spizellomyces_punctatus_DAOM_BR117
FuCh_RKO99305_Caulochytrium_protostelioides
FuCh_ORY44545_Rhizoclosmatium_globosum
FuCh_TPX73662_Chytriomyces_confervae
FuCh_TPX48556_Synchytrium_endobioticum
FuCh_XP_031022975_Synchytrium_microbalum
FuCh_KXS21829_Gonapodya_prolifera_JEL478
AKO1_000153
AKO1_009350
FuCh_ORX45236_Piromyces_finnis
AKO1_000153
AKO1_009350
FuXx_KAF0428195_Gigaspora_margarita
FuXx_RIA96111_Glomus_cerebriforme
FuXx_ORX93405_Basidiobolus_meristosporus_CBS_931.73
FuXx_ORX94057_Basidiobolus_meristosporus_CBS_931.73
FuXx_KAG0211859_Mortierella_sp._GBA30
FuXx_KAG0244954_Mortierella_sp._GBA43
FuXx_KXN73564_Conidiobolus_coronatus_NRRL_28638
FuXx_KAF7741523_Entomophthora_muscae
FuXx_RKP38903_Dimargaris_cristalligena
Pla_PXF45816_Gracilariopsis_chorda
Pla_XP_005712909_Chondrus_crispus
Pla_OSX72801_Porphyra_umbilicalis
Pla_KAA8499275_Porphyridium_purpureum
SAR_CAB1097268_Ectocarpus_sp._CCAP_1310/34
Pla_XP_005837724_Guillardia_theta_CCMP2712
FuXx_RKP16896_Rozella_allomycis_CSF55
SAR_TEB19421_Perkinsus_sp._BL_2016
Amo_XP_012755255_Acytostelium_subglobosum_LB1
Amo_XP_020432012_Heterostelium_album_PN500
Amo_XP_004351990_Cavenderia_fasciculata
Pla_KAG1676835_Chlamydomonas_sp._UWO_241
Pla_GAX80490_Chlamydomonas_eustigma
SAR_OLP82670_Symbiodinium_microadriaticum
Pla_XP_005760134_Emiliania_huxleyi_CCMP1516
Pla_XP_005821368_Guillardia_theta_CCMP2712
Ani_XP_014159878_Sphaeroforma_arctica_JP610
Pla_XP_031112474_Ipomoea_triloba
Pla_QIN90911_Eucommia_ulmoides
Pla_KAA6423855_Trebouxia_sp._A1-2
Pla_XP_005651136_Coccomyxa_subellipsoidea_C-169
Dis_KPI83893_Leptomonas_seymouri
Dis_EPY37291_Strigomonas_culicis
Dis_XP_028886513_Trypanosoma_theileri
Dis_CCW64685_Phytomonas_sp._isolate_EM1
SAR_GBG28982_Hondaea_fermentalgiana
SAR_KAA0146414_Cafeteria_roenbergensis
Amo_PRP79514_Planoprotostelium_fungivorum
Ani_XP_033099294_Anneissia_japonica
Ani_XP_031573996_Actinia_tenebrosa
Ani_XP_001750250_Monosiga_brevicollis_MX1
Ani_XP_004998350_Salpingoeca_rosetta
Fun_XP_009492725_Fonticula_alba
Ani_XP_004349424_Capsaspora_owczarzaki_ATCC_30864
Ani_XP_013760965_Thecamonas_trahens_ATCC_50062
FuCh_ORX80166_Anaeromyces_robustus
FuCh_ORX47633_Piromyces_finnis
FuCh_KXS21124_Gonapodya_prolifera_JEL478
Dis_KAF0982424_Naegleria_fowleri
Dis_XP_002683528_Naegleria_gruberi_strain_NEG-M
0.2
b) AKO1_001003
Str_KAJ8601124_Chrysophaeum_taylorii
Str_CAH0378657_Pelagomonas_calceolata
KAJ1461886_Pelagophyceae_sp._CCMP2097
Str_GMI10580_Triparma_laevis_f._longispina
Str_GMH71826_Triparma_laevis_f._inornata
Dino_GMI32097_Tetraparma_gracilis
Hapto_KAG8457749_Diacronema_lutheri
AKO1_001003
AKO1_001003
Str_CAB9503087_Seminavis_robusta
Bac_MBS0648466_Verrucomicrobiota_bacterium
Dino_CAE8711561_Polarella_glacialis
Dino_CAE8741962_Polarella_glacialis
Dino_CAE8606547_Polarella_glacialis
Hapto_KAG8461696_Diacronema_lutheri
Str_OEU09001_Fragilariopsis_cylindrus_CCMP1102
GAlg_XP_003081393_Ostreococcus_tauri
GAlg_XP_001419853_Ostreococcus_lucimarinus_CCE9901
GAlg_XP_002503697_Micromonas_commoda
Dis_KAJ9444145_Diplonema_papillatum
GAlg_KAK3235703_Cymbomonas_tetramitiformis
GAlg_KAK3235702_Cymbomonas_tetramitiformis
Disc_KAJ9461020_Diplonema_papillatum

c) AKO1_001251

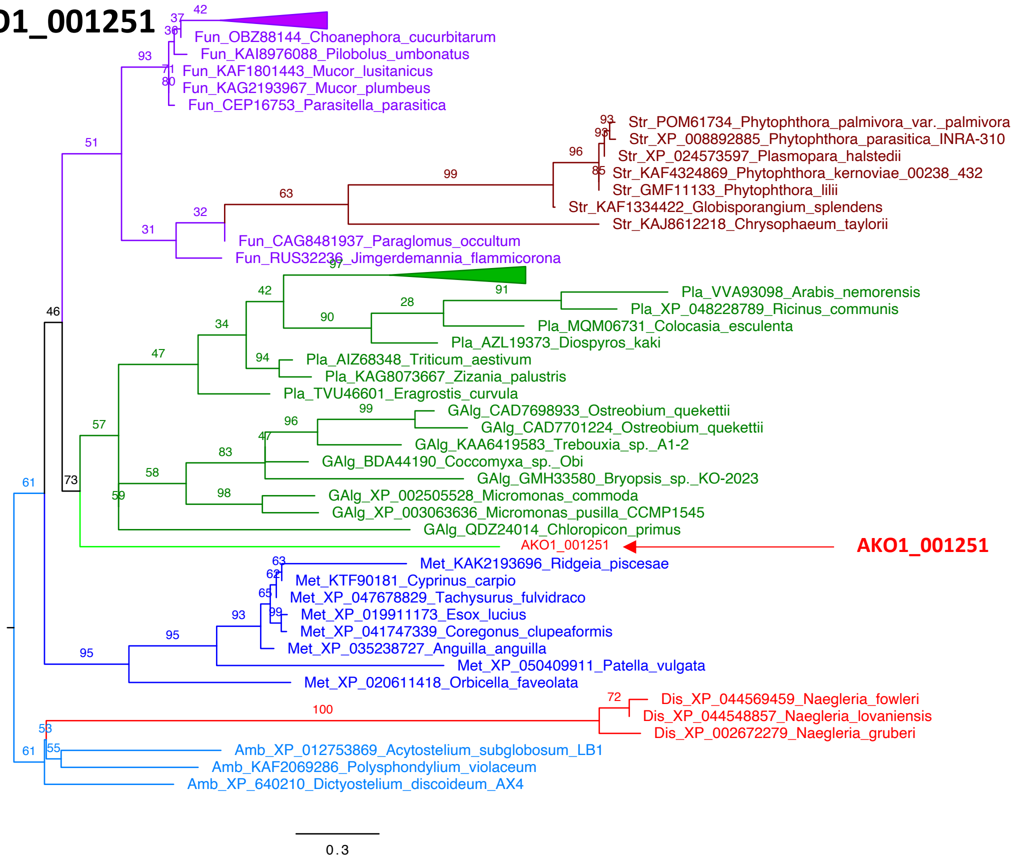

d) AKO1_001528

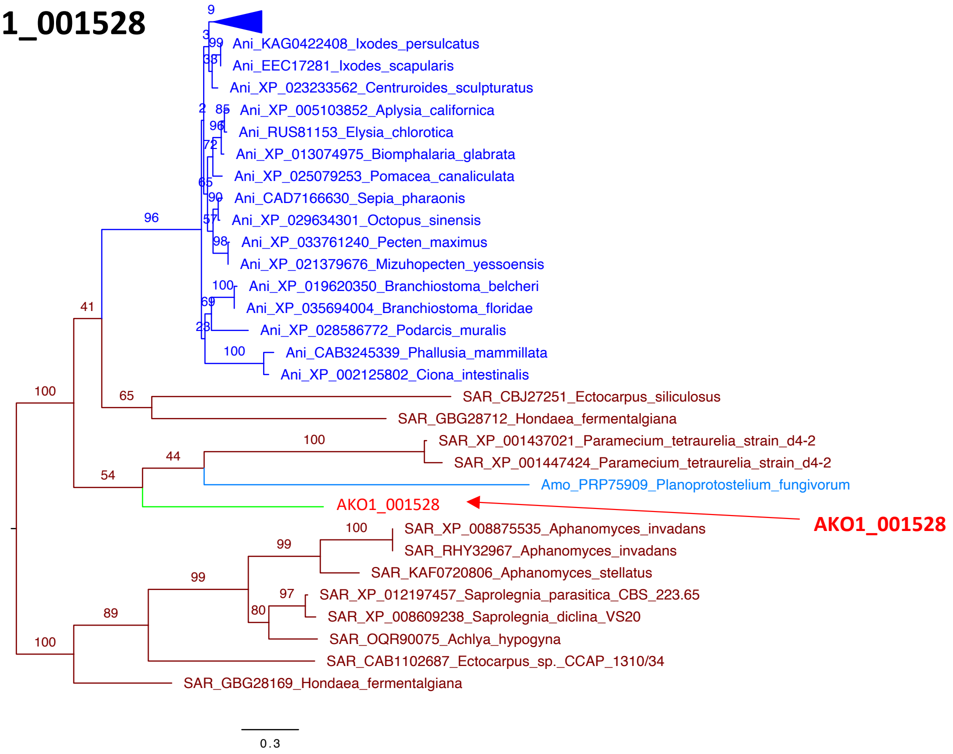

e) AKO1_002511

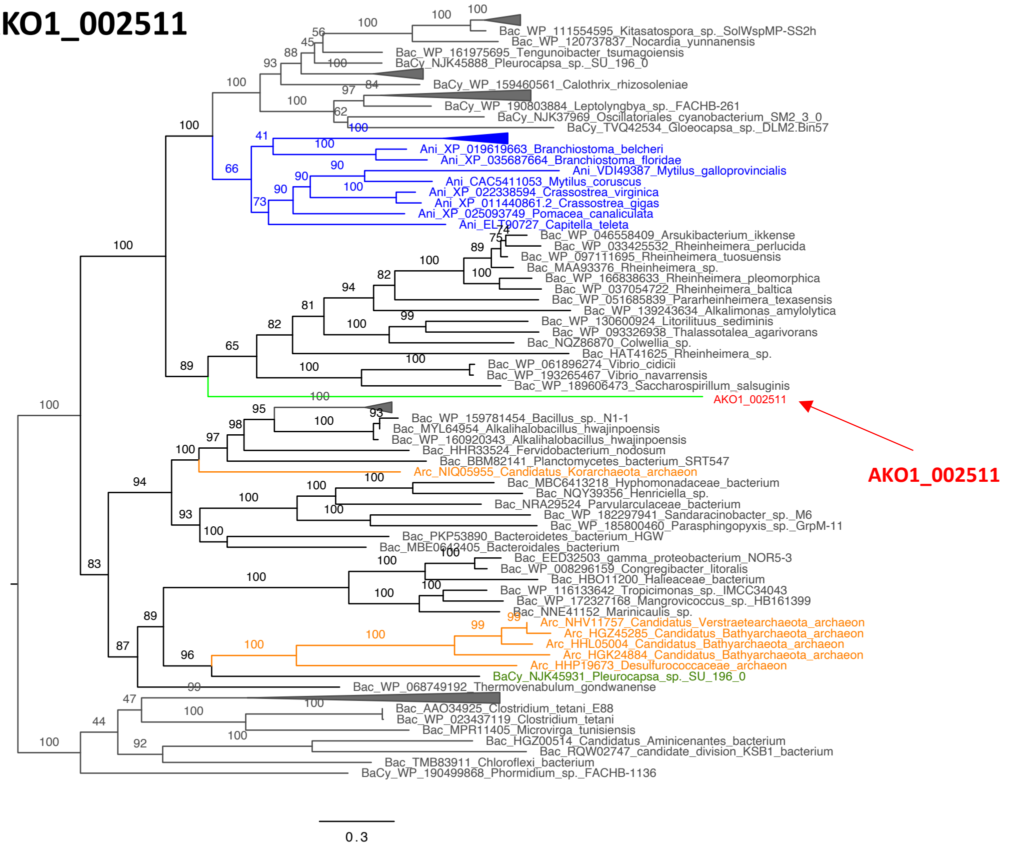

f) AKO1_002520

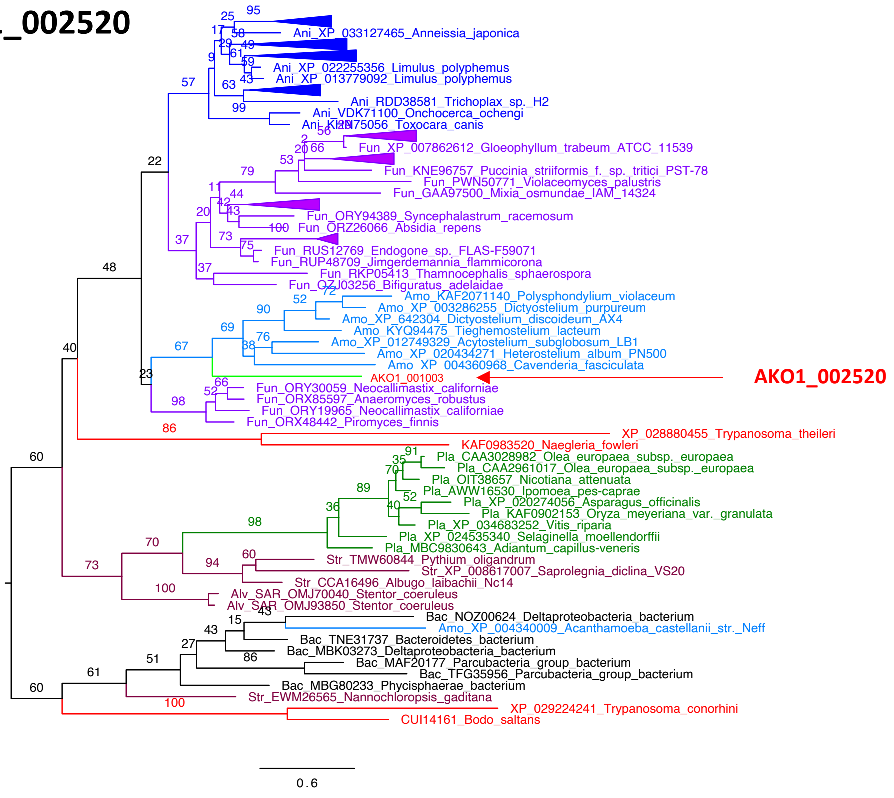

g) AKO1_002921 AKO1_012347

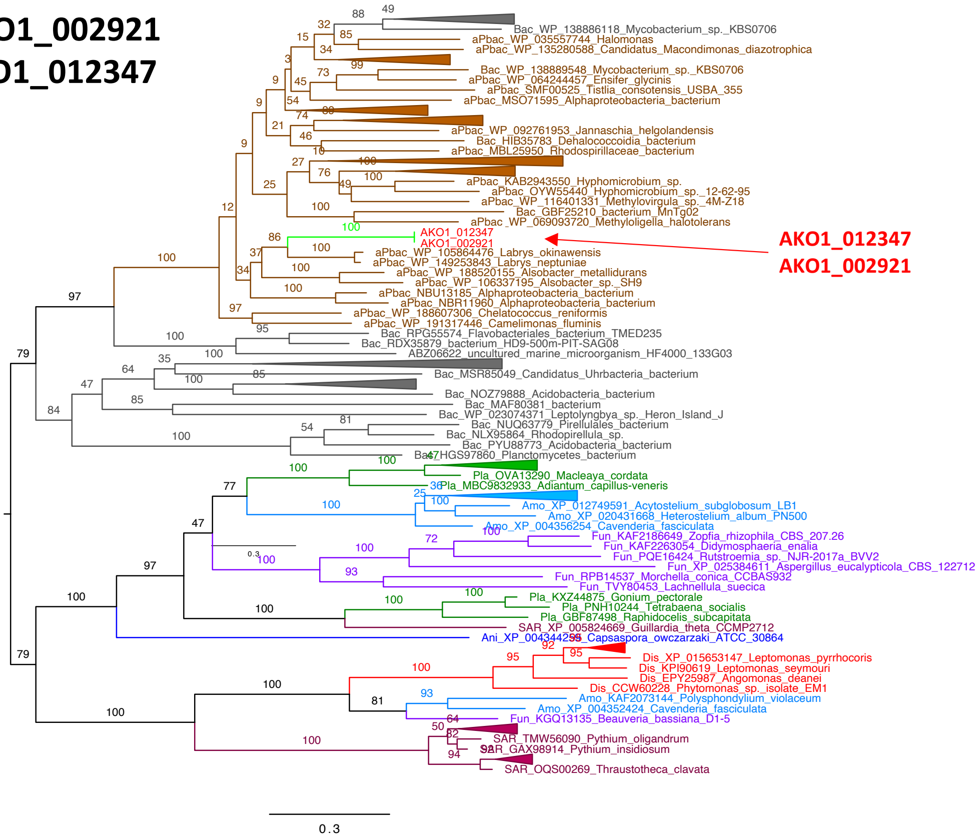

h) AKO1_002929

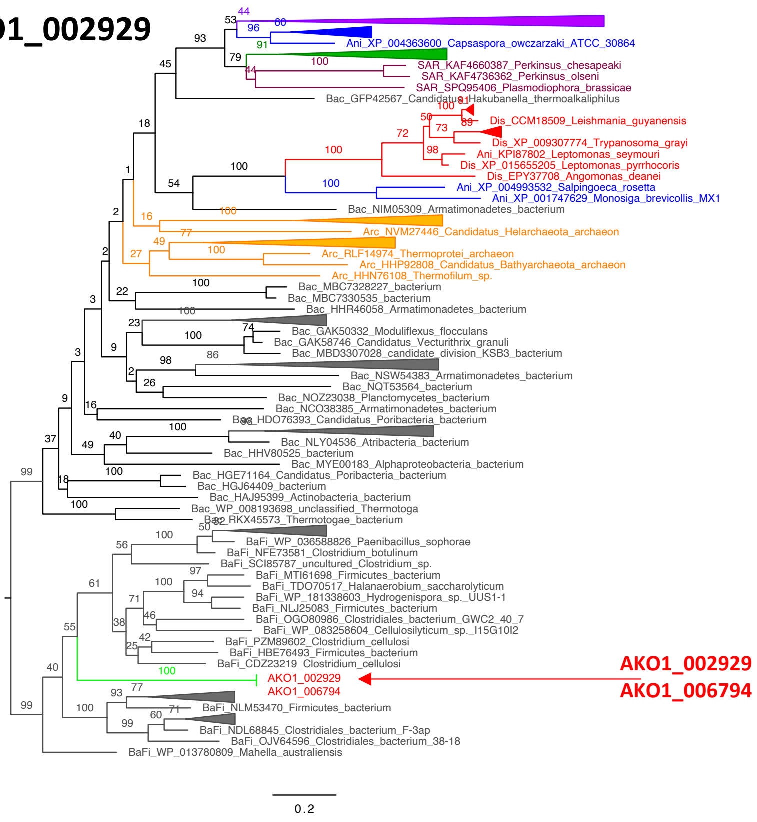

i) AKO1_004504

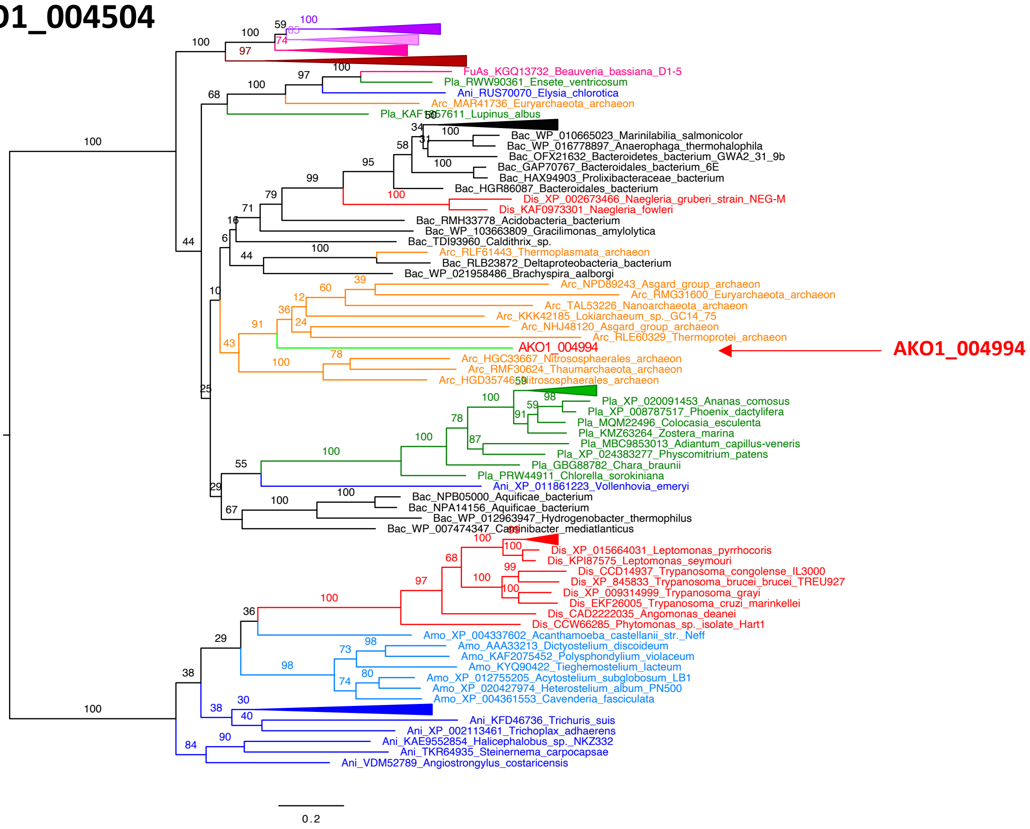

j) AKO1_004994

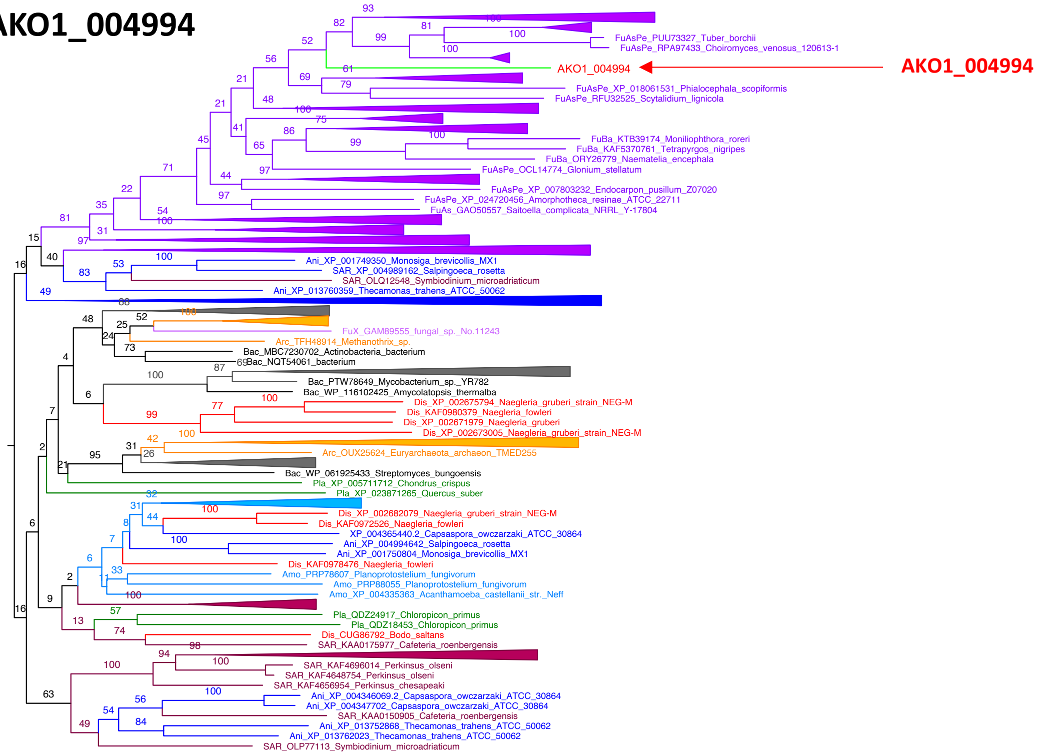

k) AKO1_006518

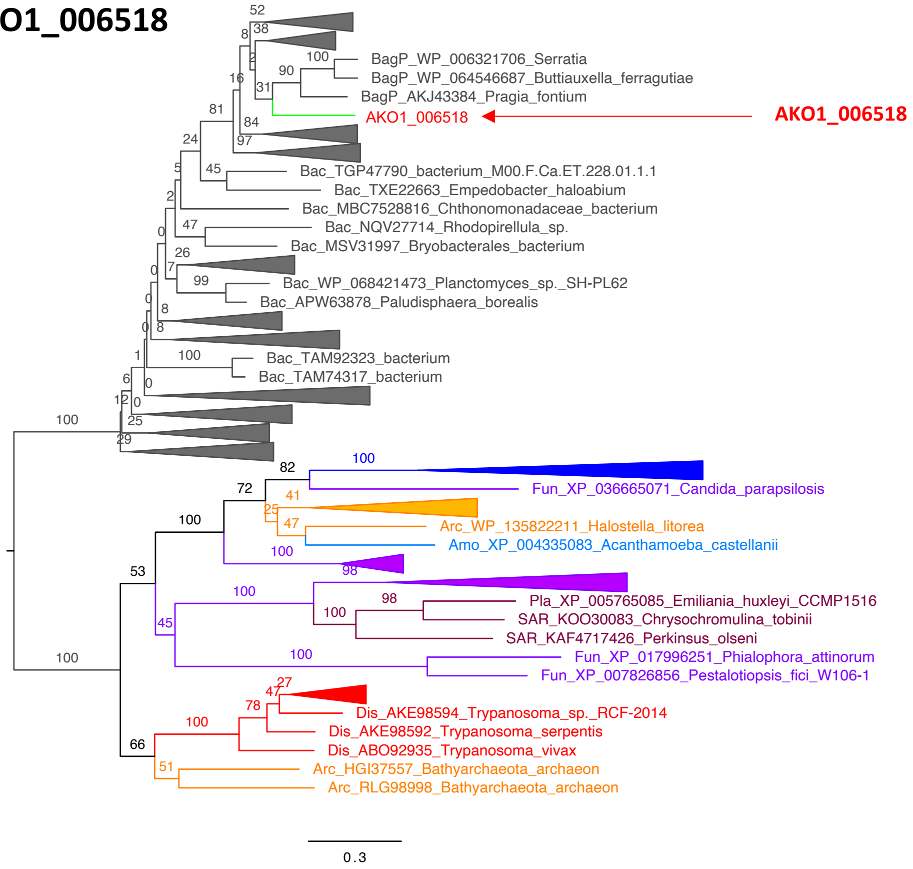

l) AKO1_007316

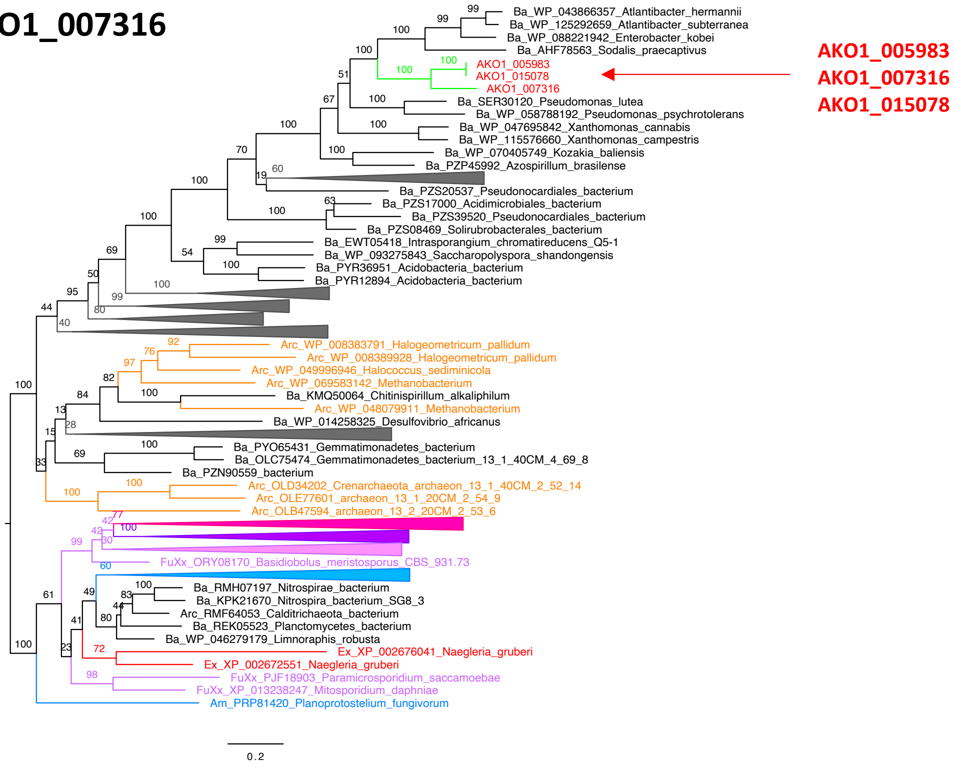

m) AKO1_008377

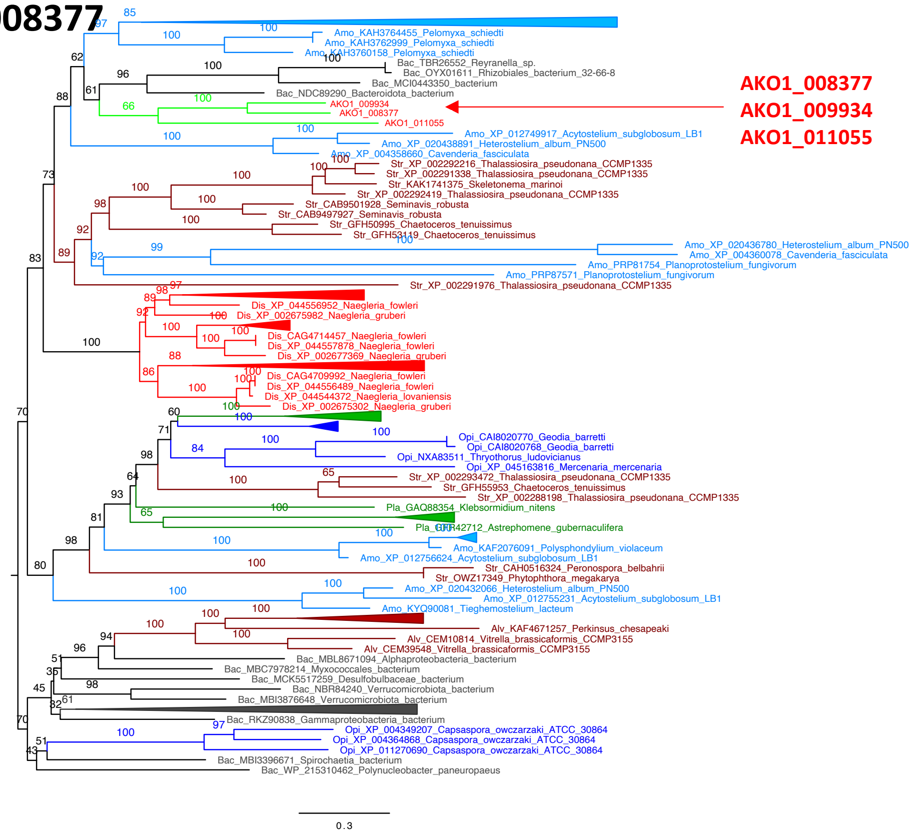

n) AKO1_008403

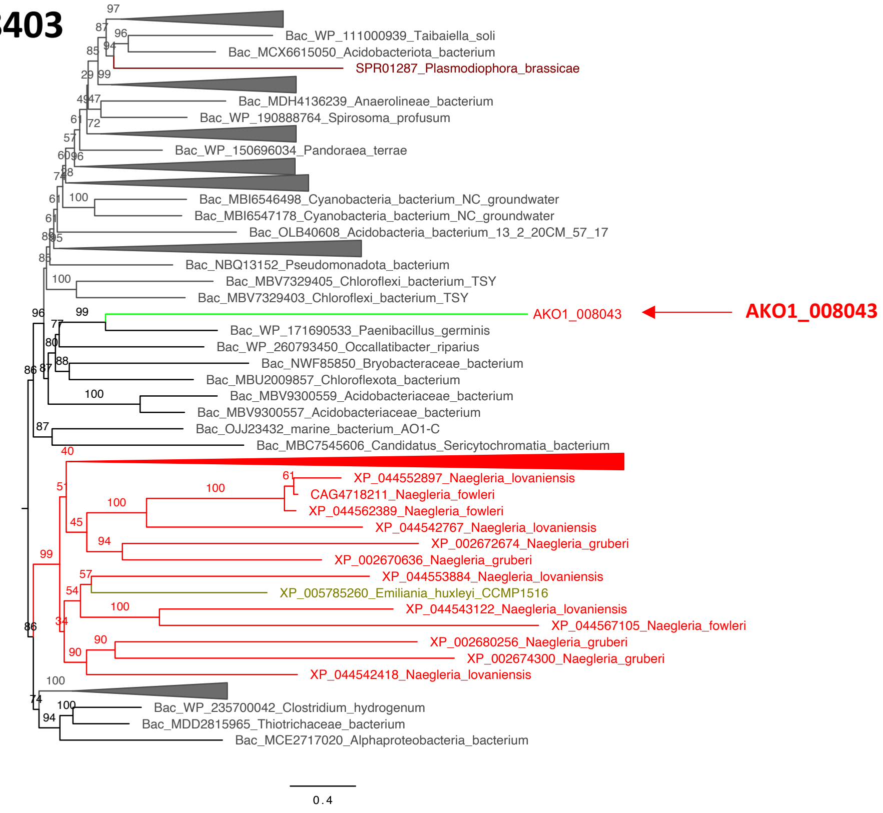

o) AKO1_008555

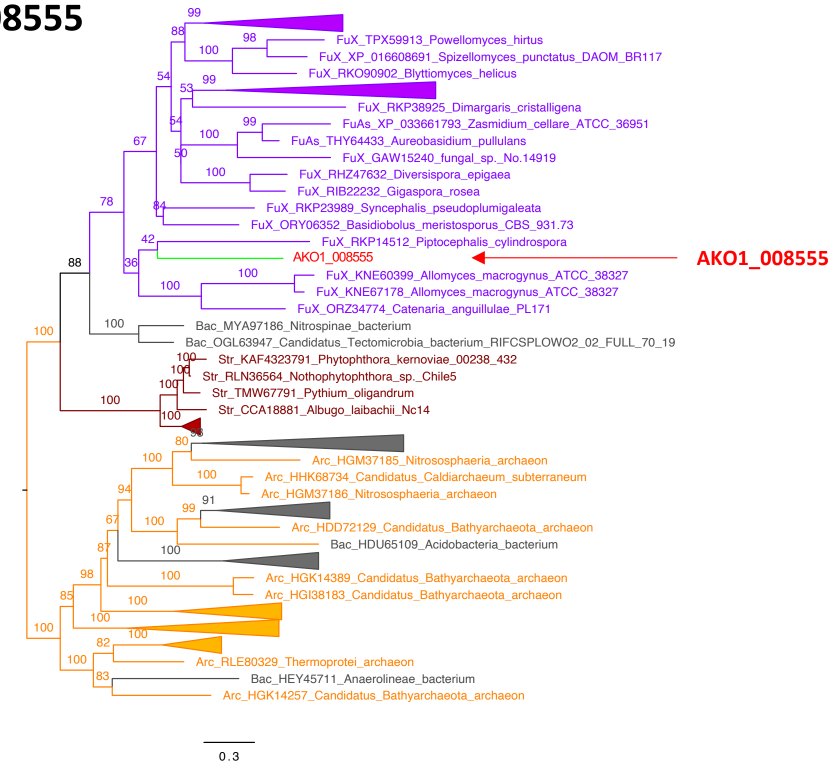

p) AKO1_008593

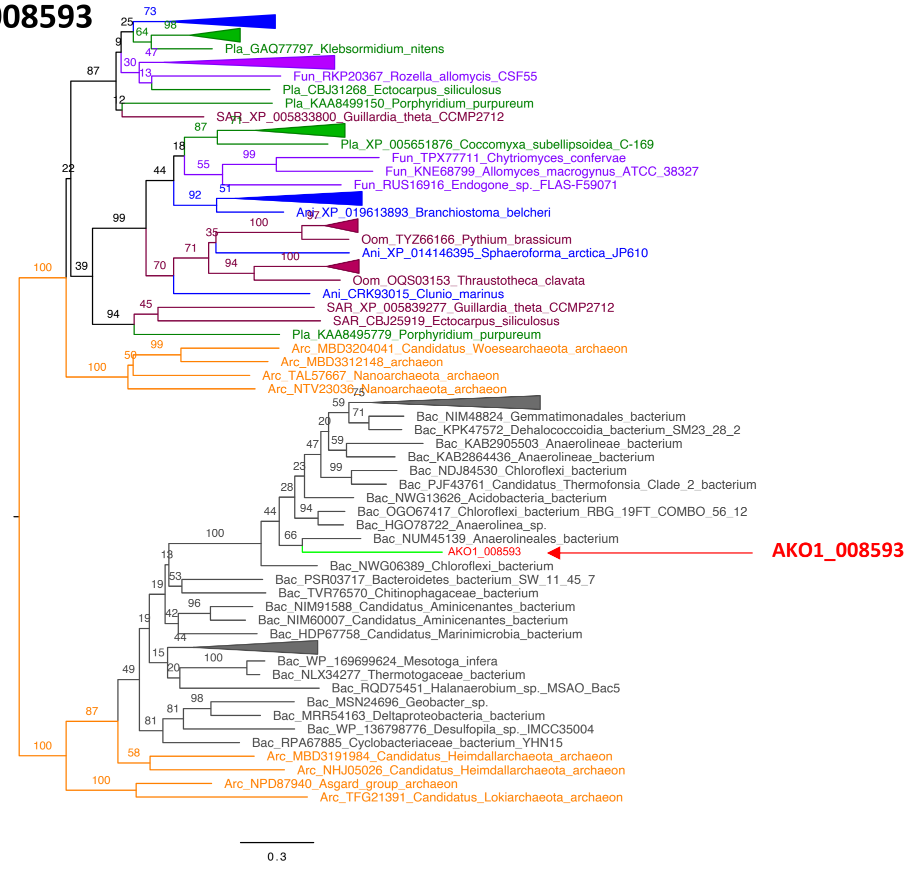

q) AKO1_008594

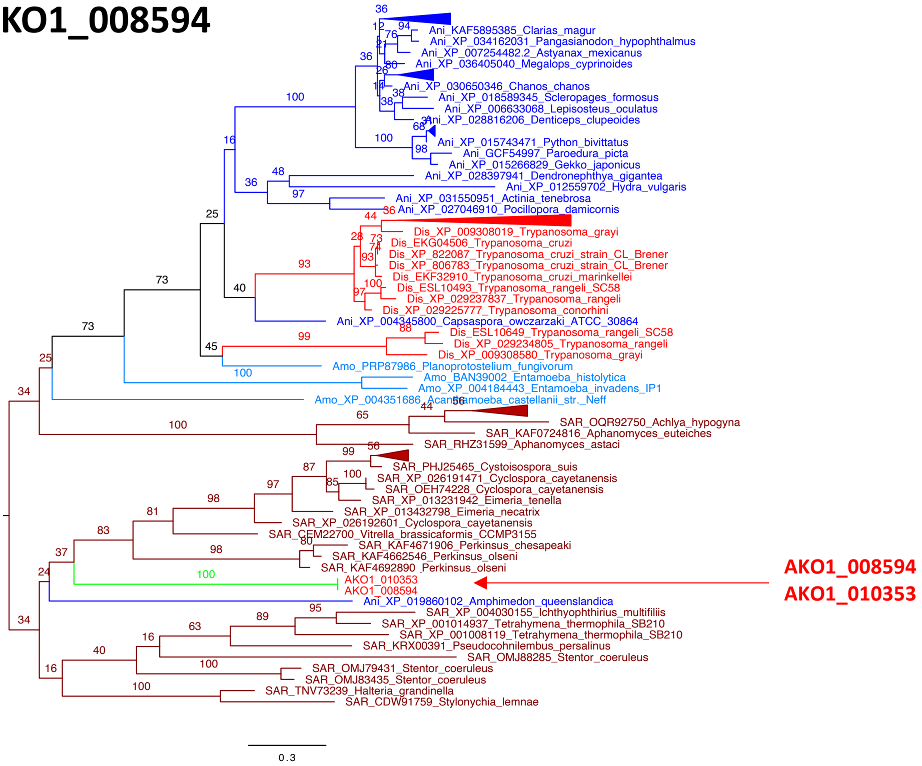

r) AKO1_009350

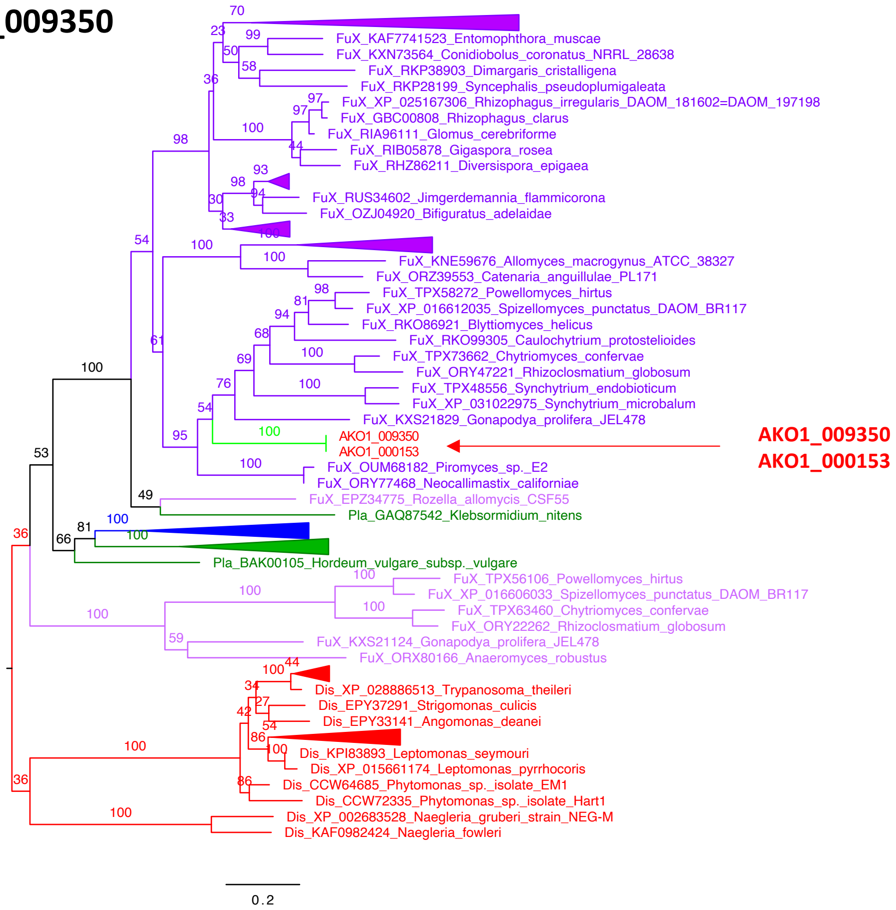

s) AKO1_009644

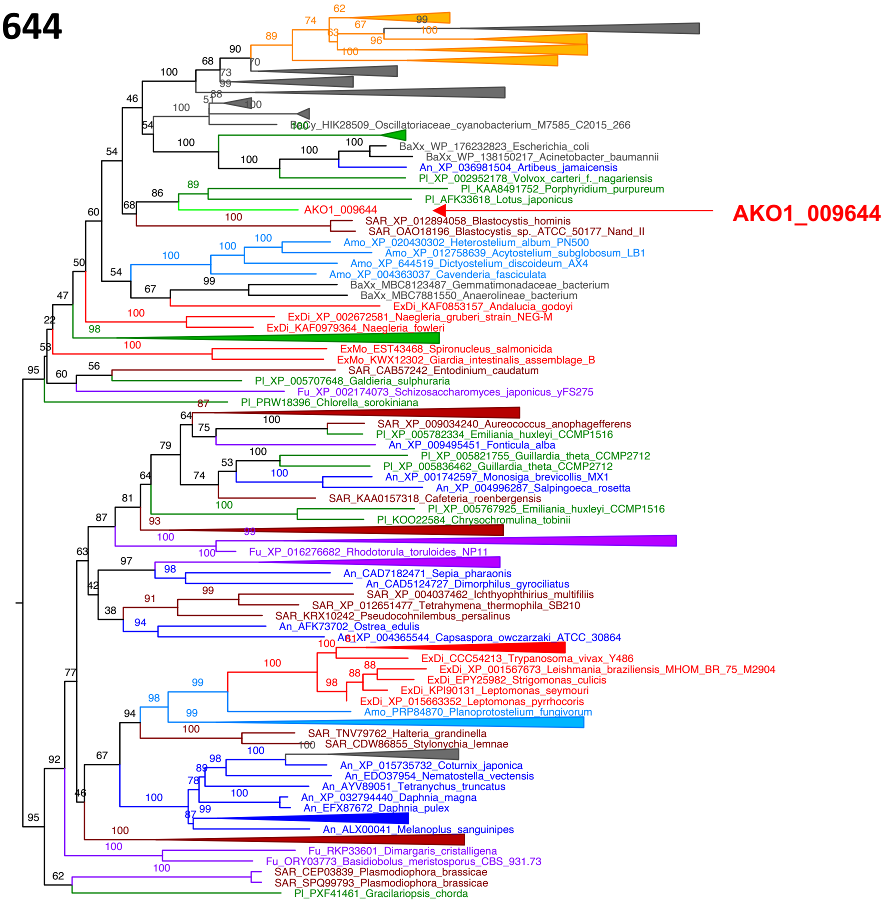

t) AKO1_011637

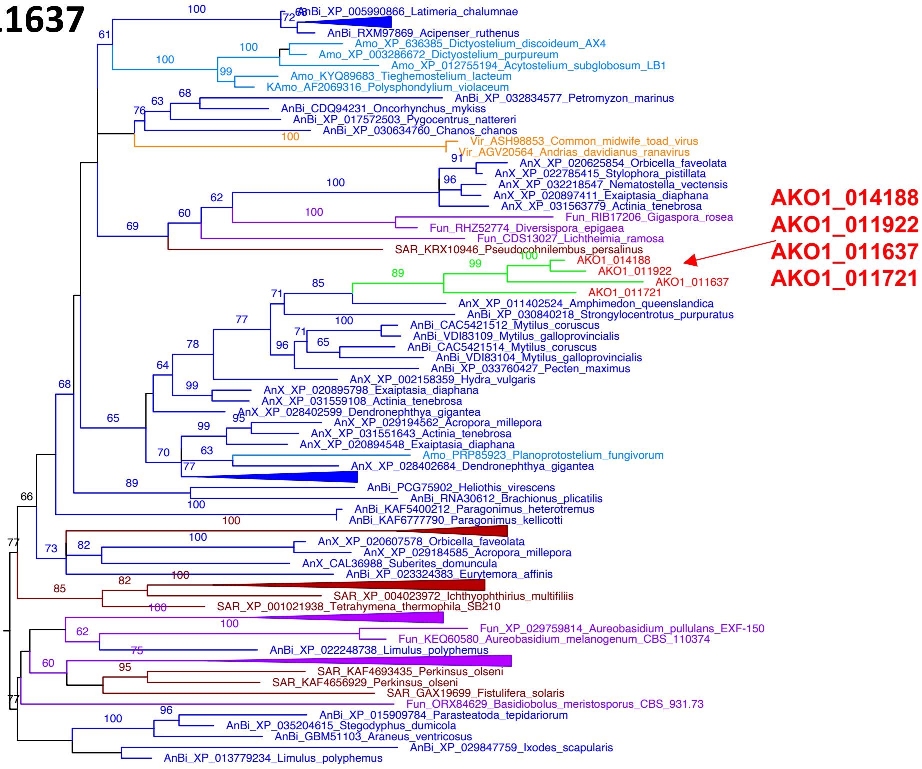

u) AKO1_011734

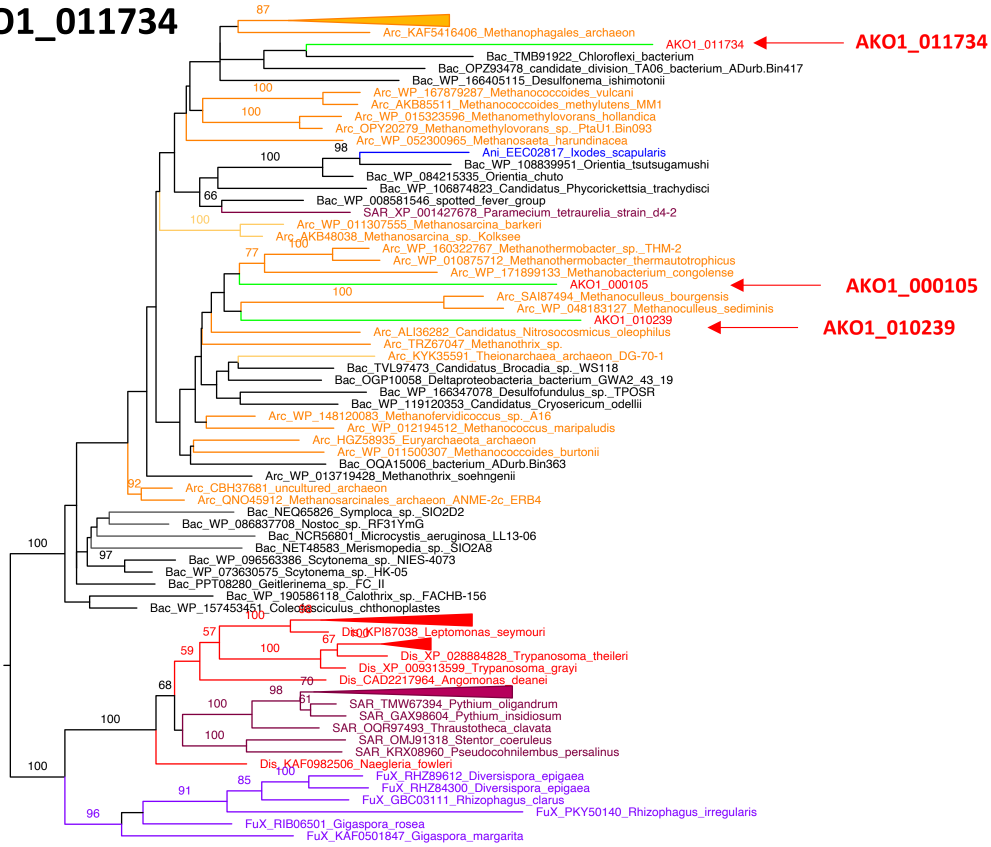

v) AKO1_011857

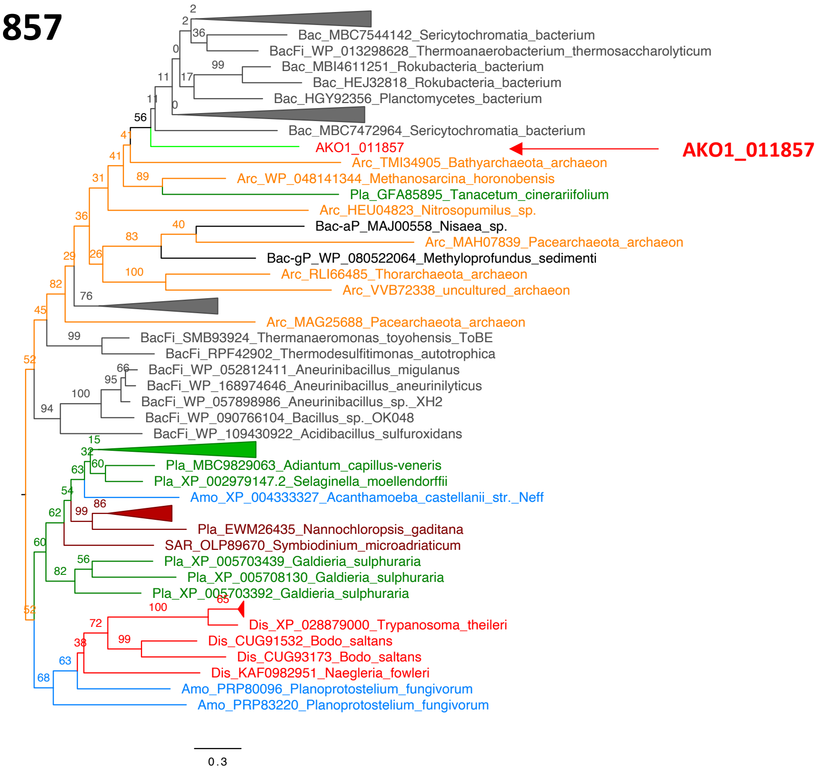

w) AKO1_011976

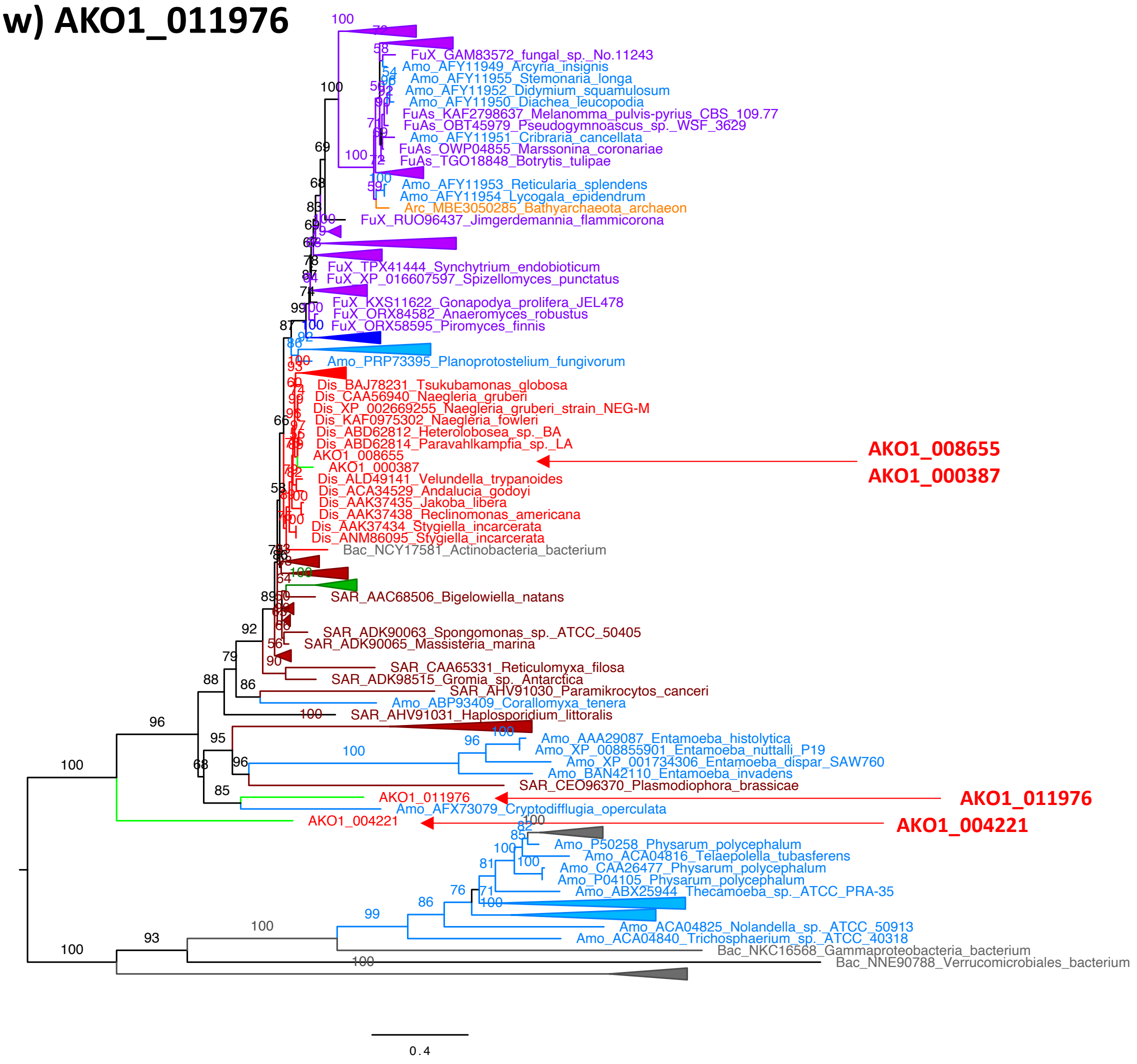

x) AKO1_012265

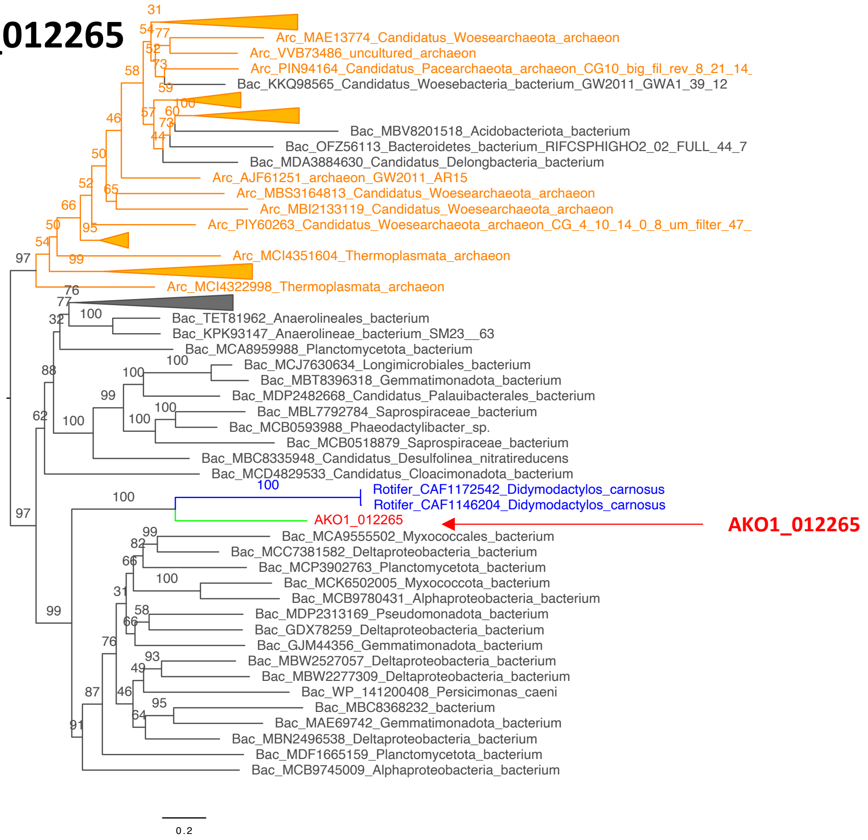

y) AKO1_013224

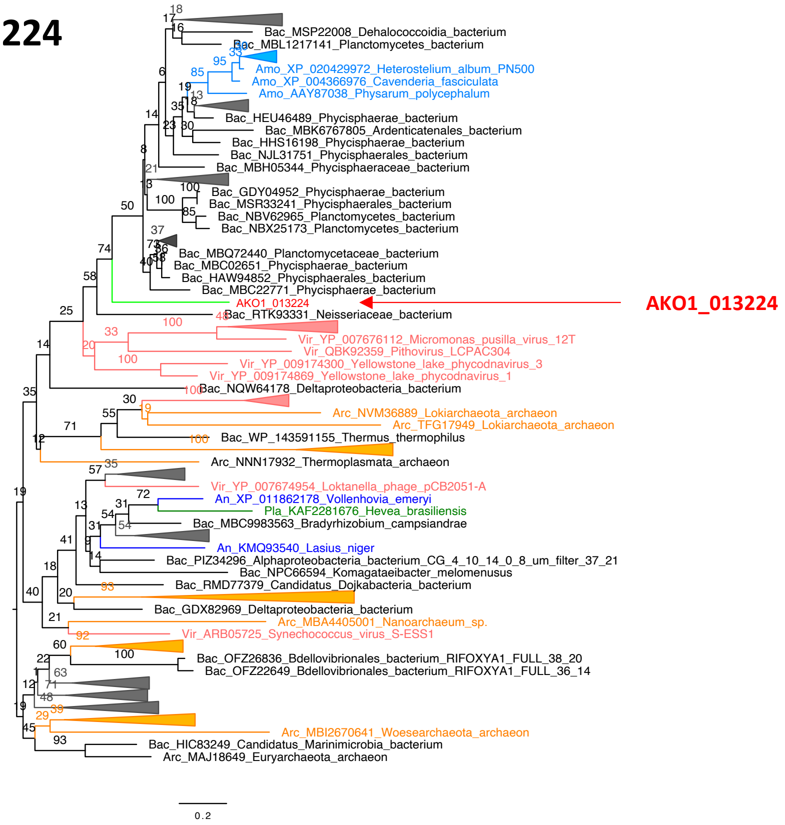

z) AKO1_013702

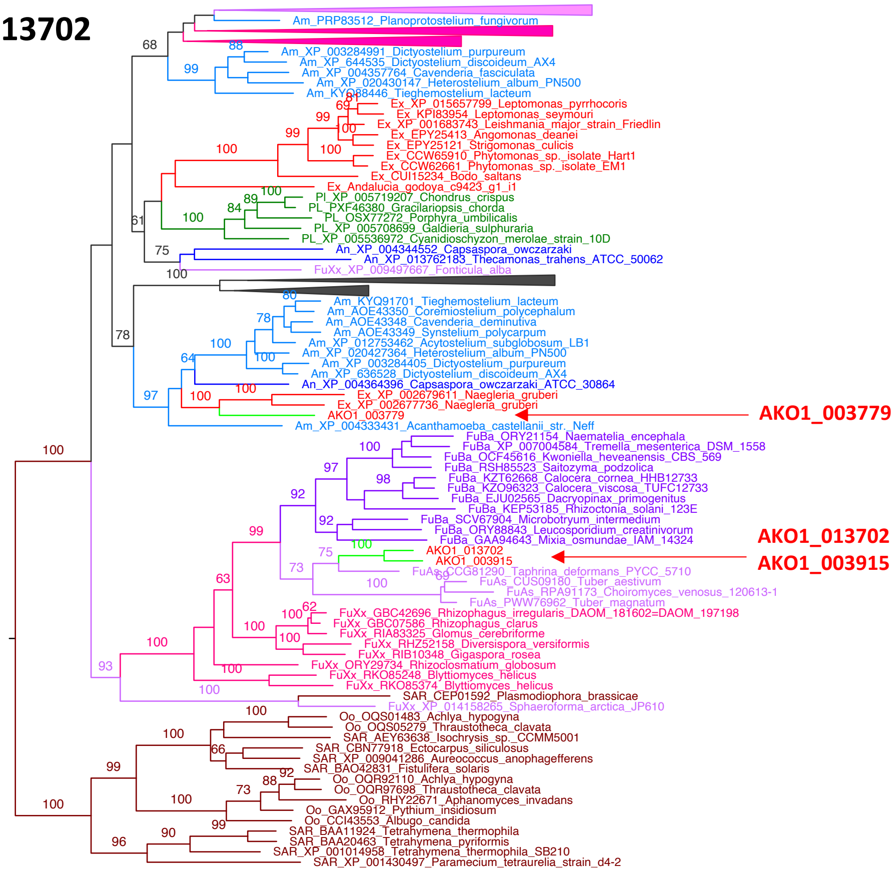

aa) AKO1_014197

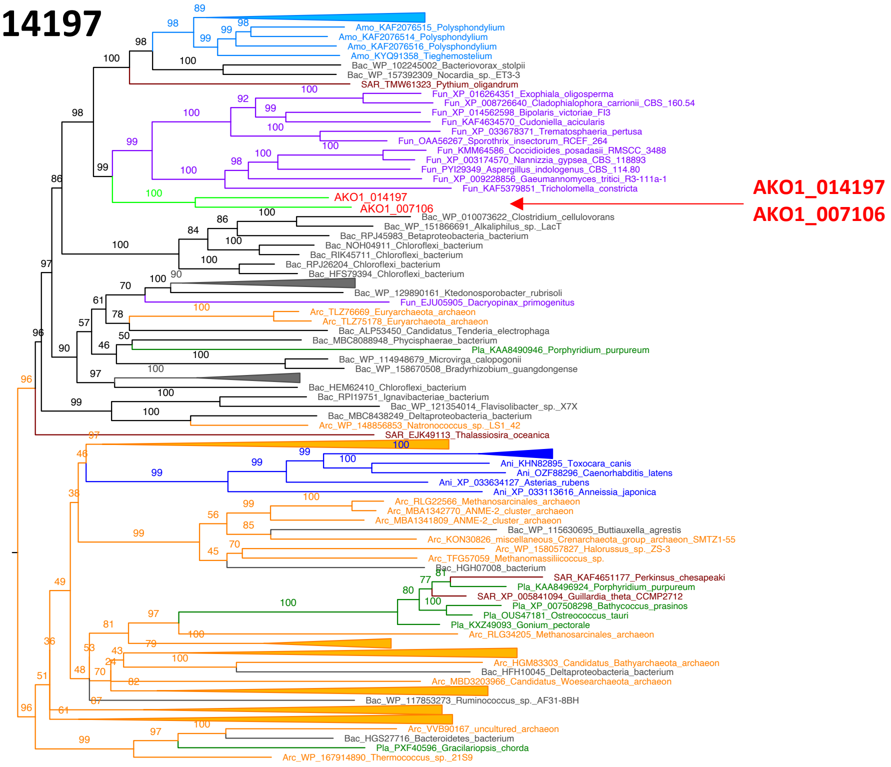

ab) AKO1_014302

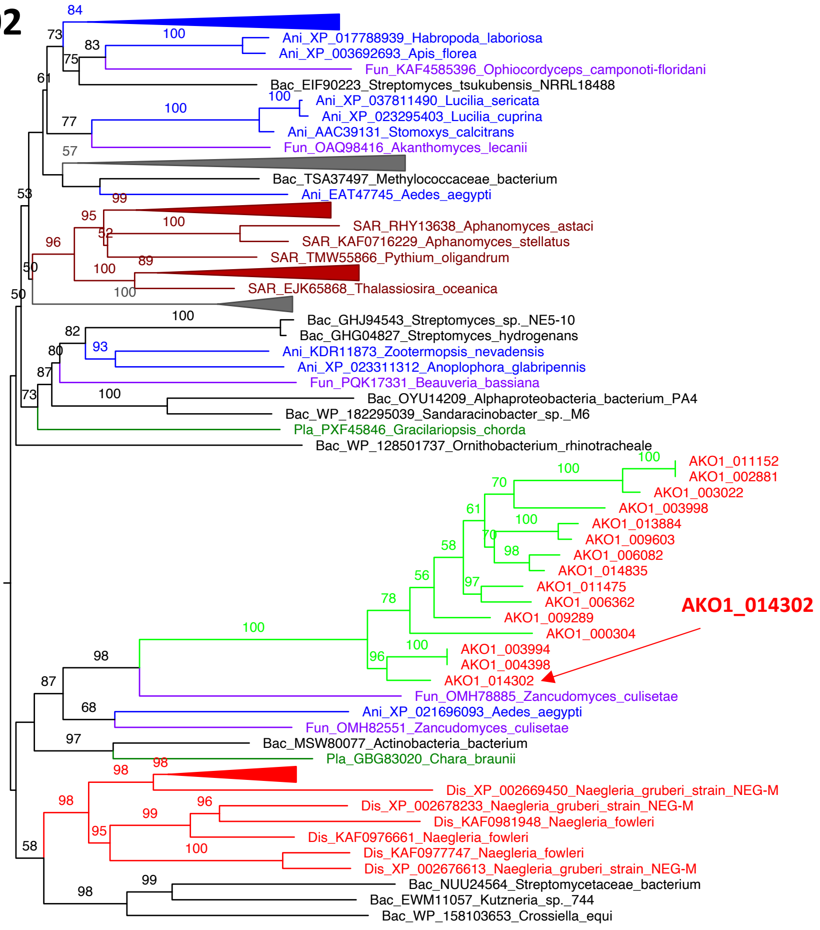

ac) AKO1_014780

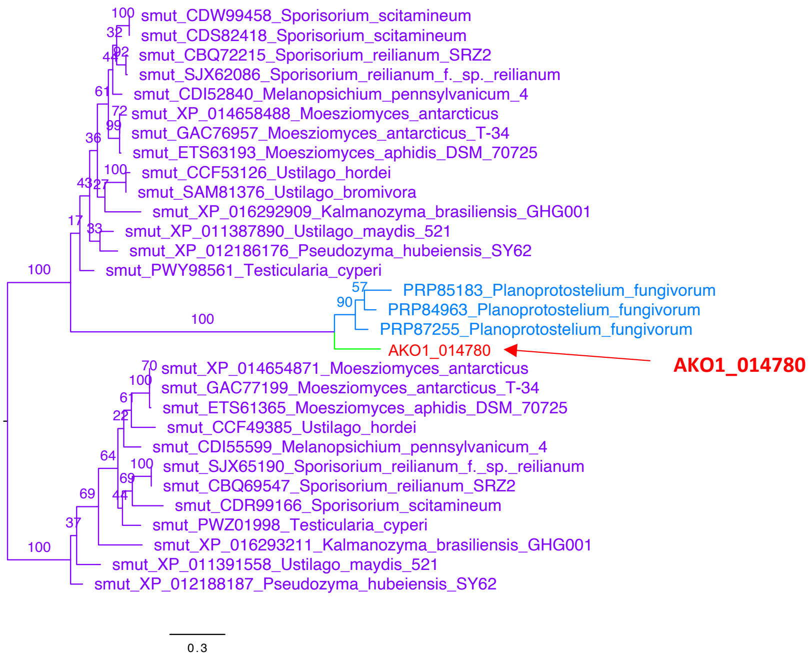

ad) AKO1_015320

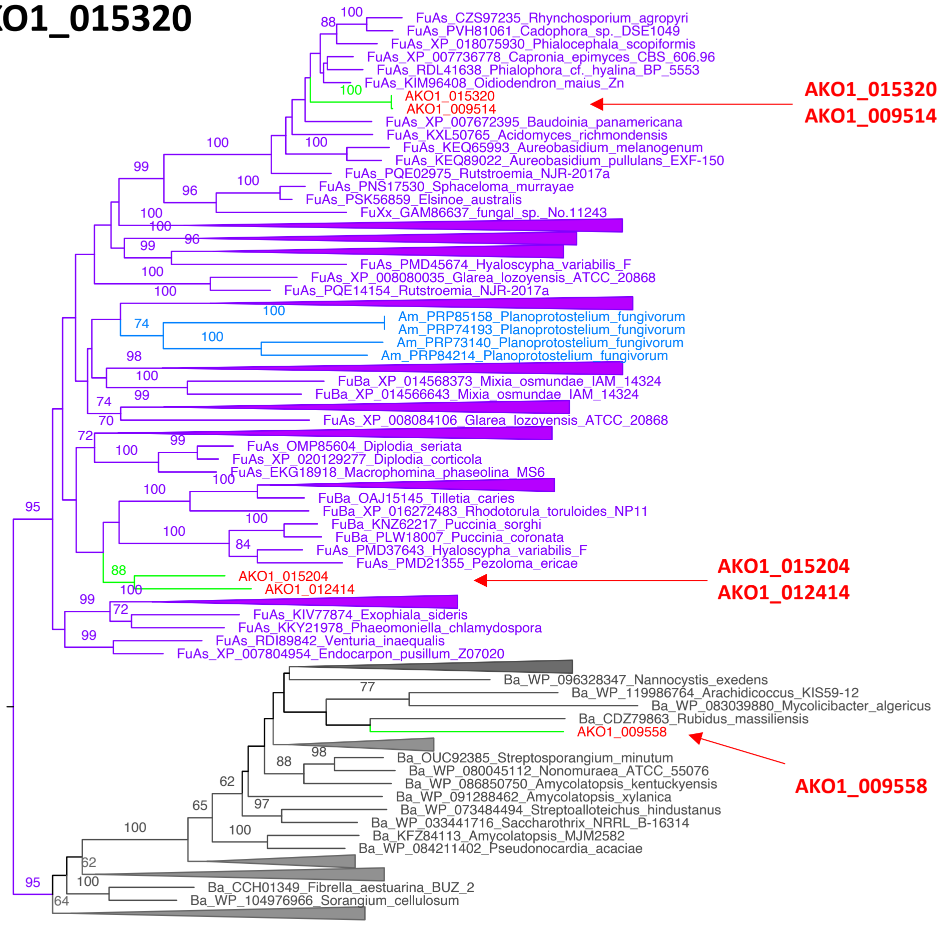

ae) AKO1_015510

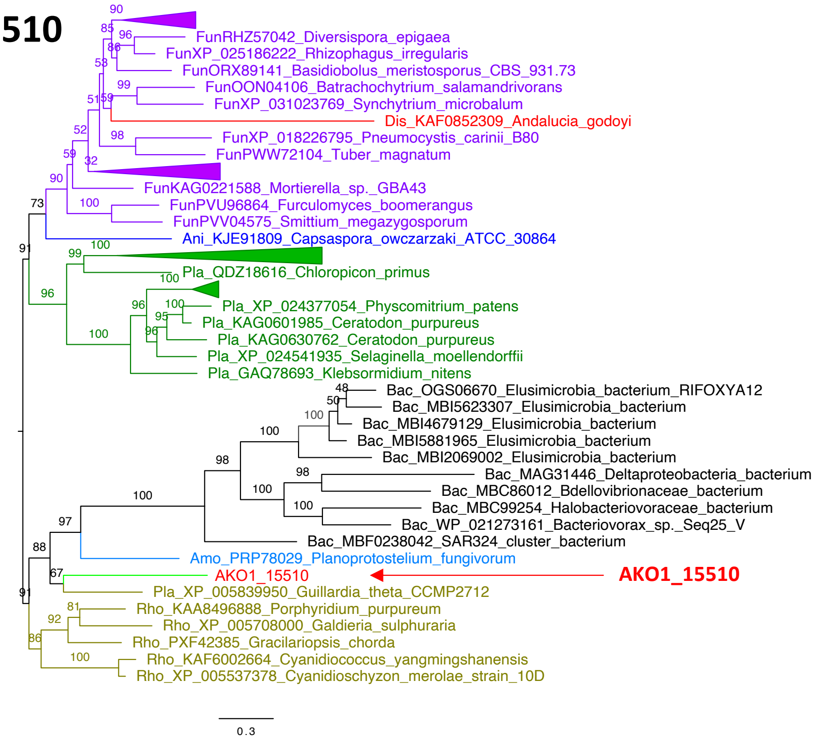

af) AKO1_015696

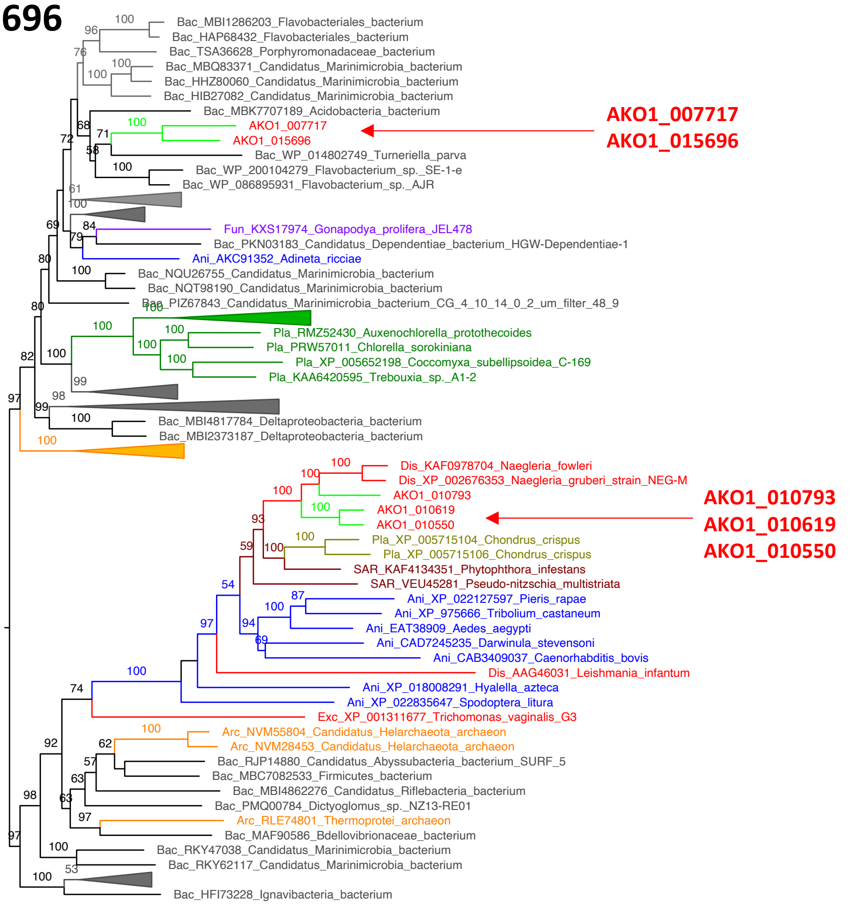

ag) AKO1_015737

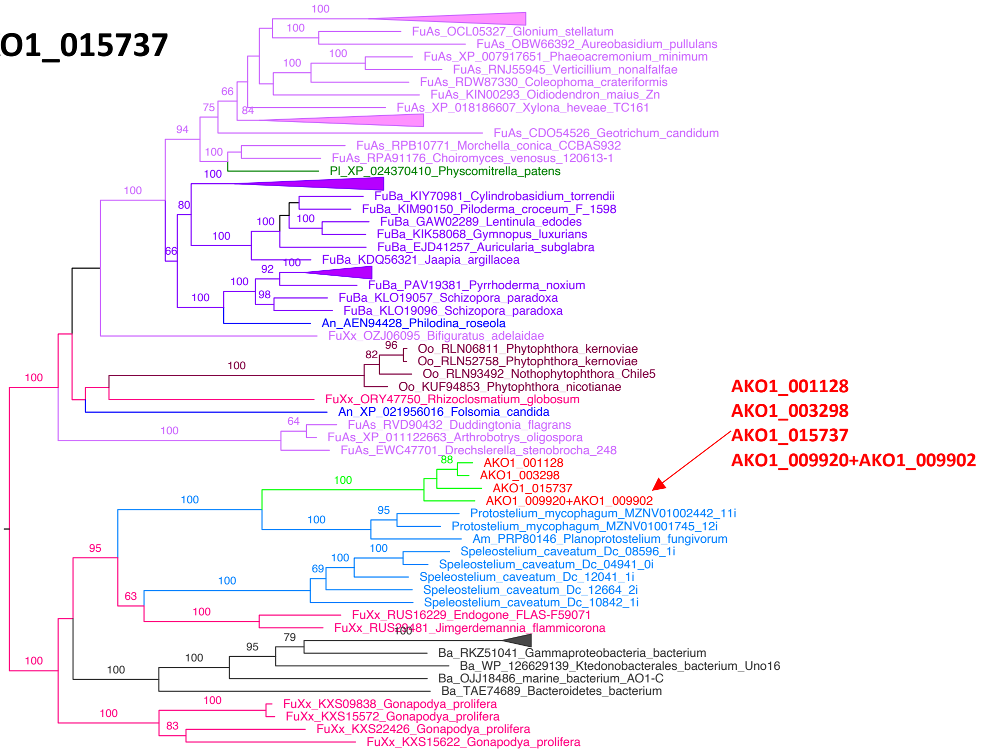

Figure S4. Phylogenetic support for *Acrasis kona* aggregation proteins showing evidence of horizontal transfer. Protein sequence phylogenies are shown for 34 *A. kona* Aggup genes showing strong evidence of evolution by horizontal gene transfer. The proteins are as follows: a) ABC ATPase (AKO1_000153), b) alpha-ketoglutarate-dependent dioxygenase (AKO1_001003), c) CCAAT-binding transcription factor subunit B (AKO1_001251), d) F-box/SPRY domain-containing protein 1 (AKO1_001528), e) glutamine amidotransferase type 1 (AKO1_002511), f) transformer-2 protein (AKO1_002520), g) SAM methyltransferase (AKO1_002921 and AKO1_012347), h) NAD(P)-dependent alcohol dehydrogenase (AKO1_002929), i) guanine synthetase (AKO1_004504), j) alpha/beta hydrolase (AKO1_004994), k) 4-hydroxyproline epimerase (AKO1_006518), l) glycoside hydrolase family 15 protein (AKO1_007316), m) AMP/GMP cyclase (AKO1_008377), n) LRE family protein (AKO1_008403), o) dimethylmenaquinone methyltransferase (AKO1_008555), p) phospholipid methyltransferase (AKO1_008593), q) lipid-binding START domain protein (AKO1_008594), r) elongation factor 3 (AKO1_009350), s) nucleoside-diphosphate kinase (AKO1_009644), t) LITAF-like protein (AKO1_011637), u) tetratricopeptide repeat protein (AKO1_011734), v) phosphatidyl-myo-inositol mannosyltransferase (AKO1_011857), w) beta tubulin (AKO1_011976), x) class I SAM-dependent methyltransferase (AKO1_012265), y) thymidylate synthase (AKO1_013224), z) stearoyl-CoA desaturase (AKO1_013702), aa) SAM-dependent methyltransferase (AKO1_014197), ab) trypsin-like serine protease (AKO1_014302), ac) salicylate 1-monooxygenase (AKO1_015320), ad) scramblase domain protein (AKO1_015510), ae) beta-glucosidase (AKO1_015696), and af) rvt reverse transcriptase (AKO1_015737). AKO1_013406 is shown in Supplementary Fig. S3. Trees were derived by maximum likelihood analysis of deduced amino acid sequences. Alignment lengths, phyogenetic methods and models used to derive the trees and tree desciptions in newick format are given in Supplementary Data 8. Trees are drawn to scale as indicated by individual scale bars and with bootstrap percentages shown on the relevant branches. Sequence labels are color-coded according to higher-level taxonomy of their host species as indicated in the key to Figure S3.

location	structure, function		protein	Acrasis accession	Human vs: Ako	Ngr	Cva	Ptr	Aan	Ddi	Sce	Spo	Acrasis development: $RPKM_{Gro}$	$RPKM_{Agg}$	$RPKM_{Gm}$	DE_{Agg}	DE_{Grm}	Dictyostelium development: $RPKM_0$	$RPKM_5$	DE_{0_1}	DE_{0_5}
universal	RNAse		RPR6	AKO1_006097									121.57	111.68	124.00			10.49	8.21	▼	
	core (Exo9)	S1-KH cap	Csl4	AKO1_008302									62.52	304.71	100.54	▲	▼	29.33	6.64	▼	▼
			RPR4	AKO1_008490									9.44	4.34	5.84			29.08	6.22	▼	▼
			RPR40	AKO1_002427									34.61	45.07	28.13			13.48	4.11		▼
				AKO1_009239									20.28	22.40	23.98						
		PH ring	MTR3	AKO1_007336									20.08	16.56	6.92			31.71	5.83		▼
			Rpr41/ski6	AKO1_003026									10.25	11.82	6.92			28.15	6.24		▼
				AKO1_002663									18.21	18.09	12.78						
			RPR42	AKO1_015015									13.70	15.64	8.23			23.77	3.05		▼
				AKO1_003267									17.12	10.71	5.19						
			RPR43	AKO1_009379									6.11	3.16	4.36			14.50	1.37	▼	▼
			RPR45	AKO1_014128									43.30	49.90	37.56			13.47	5.26	▼	▼
			RPR46	AKO1_014924									88.50	88.63	203.14		▲	14.39	2.71	▼	▼
nucleus	RNAse		Dis3/RPR44	AKO1_011571									35.11	41.70	35.33			13.04	2.71	▼	▼
	helicase		Mtr4	AKO1_007891									2.98	8.41	6.74	▲		16.26	2.93	▼	▼
				AKO1_001502									5.78	15.64	13.20	▲					
cytoplasm	RNAse		Dis3L/RPR44	AKO1_009867									4837.59	4807.24	4852.46			1.90	2.30		
																		7.61	10.40	▲	
	Ski complex	helicase	Ski2	AKO1_015556										8.60	0.87		▼	9.36	14.93		
		coupling	ski7/HBS1-like	AKO1_007586										28.73	19.22			46.50	96.25		▲
				AKO1_002870										11.81	0.77		▼				
		HBS1 inter	Dom34/Pelota	AKO1_009893										37.73	1.33		▼	50.22	51.15		
	RNAi	dicer	dsRNA RNAse	AKO1_013723									10.45	6.85	10.37			7.15	24.79		▲
				AKO1_008903									14.71	7.12	10.60						
		TAF11	TATA factor 11	AKO1_005317									17.76	13.89	10.26			8.25	14.29		
		RdRP	RNA-RNA pol	AKO1_005852									134.68	119.76	281.16		▲	0.53	3.98	▲	▲
				AKO1_007172									22.91	13.72	2.00		▼	1.21	2.99		
																		1.40	2.72	▲	▲
		argonaut	RISC RNAse																		
		piwi	RISC RNAse	AKO1_008445									58.08	81.45	72.37			10.26	1.68	▼	▼
				AKO1_004633									144.98	134.38	137.92			21.15	101.79		▲
																		3.27	0.83	▼	▼
																		1.05	0.54	▼	▼

BLASTp e-value
0-100
99-70
69-40
39-25
24-10, cvg> 50%
<10 / annotation

Figure S5. Sequence conservation and developmental expression of the *Acrasis kona* exosome. Homologs of exosomal proteins were identified by BLASTp using human queries. The strength of BLASTp hits (c-value) is indicated by color intensity (key at right). BLASTp hits with high e-values (e^{-24} to e^{-10}) were scored as present only if their BLASTp alignments included >50% query coverage (cvg). Exosomal components annotated as present but with only very weak BLASTp scores (> e^{-10}) are indicated in pale yellow. RNAseq length-corrected read numbers (RPKM) are shown for actively growing ($RPKM_{Gro}$), aggregating ($RPKM_{Agg}$) and germinating ($RPKM_{Germ}$) *A. kona* cells and similarly for growth and aggregation in *Dictyostelium discoideum AX4* ($RPKM_0$ and $RPKM_5$, respectively). Green and red arrows indicate substantially increased or decreased (DE >0.9 or DE <-0.9, respectively) gene expression for growth vs aggregation (DE_{Agg}) and aggregation vs germination (DE_{Germ}) in *A. kona* and similar time points for Ddi AX4, *i.e.,* 0 vs 1 (DE_{0_1}) and 0 vs 5 (DE_{0_5}) hours of starvation. Accession numbers, BLASTp e-values and gene expression values are given in Supplemental Data 11. Taxon names are abbreviated as follows: *A. kona* (Ako), *Aureococcus anophagefferens* (Aan), *Chlorella sp.* (Cva), *Ddi AX4* (Ddi), *Naegleria sp.* (Ngr), *Phaeodactylum tricornutum* (Ptr), *Saccharomyces cerevisiae S288c* (Sce) and *Schizosaccharomyces pombe* (Spo). Ddi AX4 gene expression values were derived from (Santhanam et al. 2015).

gene	Ako accession	e-values: Discoba Ako	Discoba Ngr	Str Aan	Pla Cva	Amo Ddi	Fungi Sce	Fungi Spo	development (RNAseq): Ako DE Gr:Ag	Ako DE Ag:Gm	Ako RPKM Ag	Ddi DE 0:1 hr	Ddi DE. 1:2 hr	Ddi RPKM av hr1,2
anaphase promoting complex														
Apc1	AKO1_009415									-0.47	8.55	5.35	2.28	0.48
Apc2	AKO1_001014										20.46	2.76		1.80
Apc3	AKO1_005309									-1.09	44.02			7.09
Apc5	AKO1_009574										14.19	-2.91		2.92
Apc6	AKO1_013416									-1.71	40.05			6.32
Apc8	AKO1_014201								1.03	-3.77	33.54	4.20		18.38
Apc10	AKO1_012692										0.0	-1.56	2.72	4.79
Apc11	AKO1_013097									-1.30	24.45			63.28
	AKO1_001639								-1.56		1.12			979.01
	AKO1_009363								-1.03		7.04			
centromere														
Aurora	AKO1_001925									-1.24	13.12			66.78
	AKO1_003728								2.19	-1.05	9.58			
Incenp	AKO1_013745								3.29	-1.71	9.40			9.76
CenpA	AKO1_000547								-1.31	2.63	0.18	1.60		3.76
	AKO1_002433										0			
	AKO1_003408										9.11			
	AKO1_013776										1.01			
CenpE	AKO1_013279									1.21	4.43			4.25
**inner kinetochore**														
Plk1	AKO1_005408										0	-1.47		11.16
	AKO1_008166								2.94	-1.82	43.68			
Skp1	AKO1_005396								-1.74		1.86	-1.20	3.23	1035.69
	AKO1_009381								-1.49		0.62			
outer kinetochore														
Bub3	AKO1_009515									-0.87	13.86	-0.89		62.81
												-2.15	2.00	54.11
Cdc20	AKO1_003968								1.33		8.68	-1.08	3.44	100.30
	AKO1_011901								-1.22	2.13	1.57			
Cdh1													4.35	12.01
HORMAD	AKO1_007587								-1.27	-3.31	3.43			
Mad1	AKO1_010418										23.29	1.60		2.70
Mad2	AKO1_012843								1.36	-1.09	28.79			
MadBub	AKO1_004791										4.93			15.99
	AKO1_010725										4.27			
Mps1	AKO1_015471								0.97		17.18	-2.78		4.91
RINT1	AKO1_006546									-1.81	15.05	-3.44	5.22	3.51
TRIP13	AKO1_004538									-1.25	9.94	3.75	1.33	10.94
nuclear pore proteins														
Nup107	AKO1_007456										0	1.96		7.70
Nup160	AKO1_007444										101.56	2.23		2.43

BLAST e-value
0-100
99-70
69-40
39-25
24-10, cvg>50%
24-10, annotation

Figure S6. The *Acrasis kona* kinetochore and life cycle-stage specific expression patterns. Homologs of kinetochore proteins were identified by BLASTp using *Homo sapiens* sequences as queries. The strength of BLASTp hits (c-value) is indicated by color intensity according to the key at the top right. BLASTp hits with high e-values (e^{-24} to e^{-10}) were scored as present only if their BLASTp alignments included >50% query coverage (cvg). RNAseq length-corrected read numbers (RPKM) are shown for *A. kona* for actively growing (RPKM$_0$) and aggregating (RPKM$_5$) cells and for similar time points for *Dictyostelium discoideum AX4* (growth: RPKM$_0$, aggregation: RPKM$_5$). Green and red arrows indicate substantially increased or decreased gene expression (DE >0.9, DE <-0.9, respectively) for both *A. kona* and *D. discoideum* for growth vs aggregation (DE$_{0_5}$). Accession numbers, BLASTp e-values and gene expression values are given in Supplementary Data 13. Taxon names are abbreviated as follows: *A. kona* (Ako), *Aureococcus anophagefferens* (Aan), *Chlorella variabilis* (Cva), *D. discoideum AX4* (Ddi), *Naegleria gruberi* (Ngr), and *Saccharomyces cerevisiae S288c* (Sce). *D. discoideum AX4* gene expression values were derived from Santhanam et al. (2015).

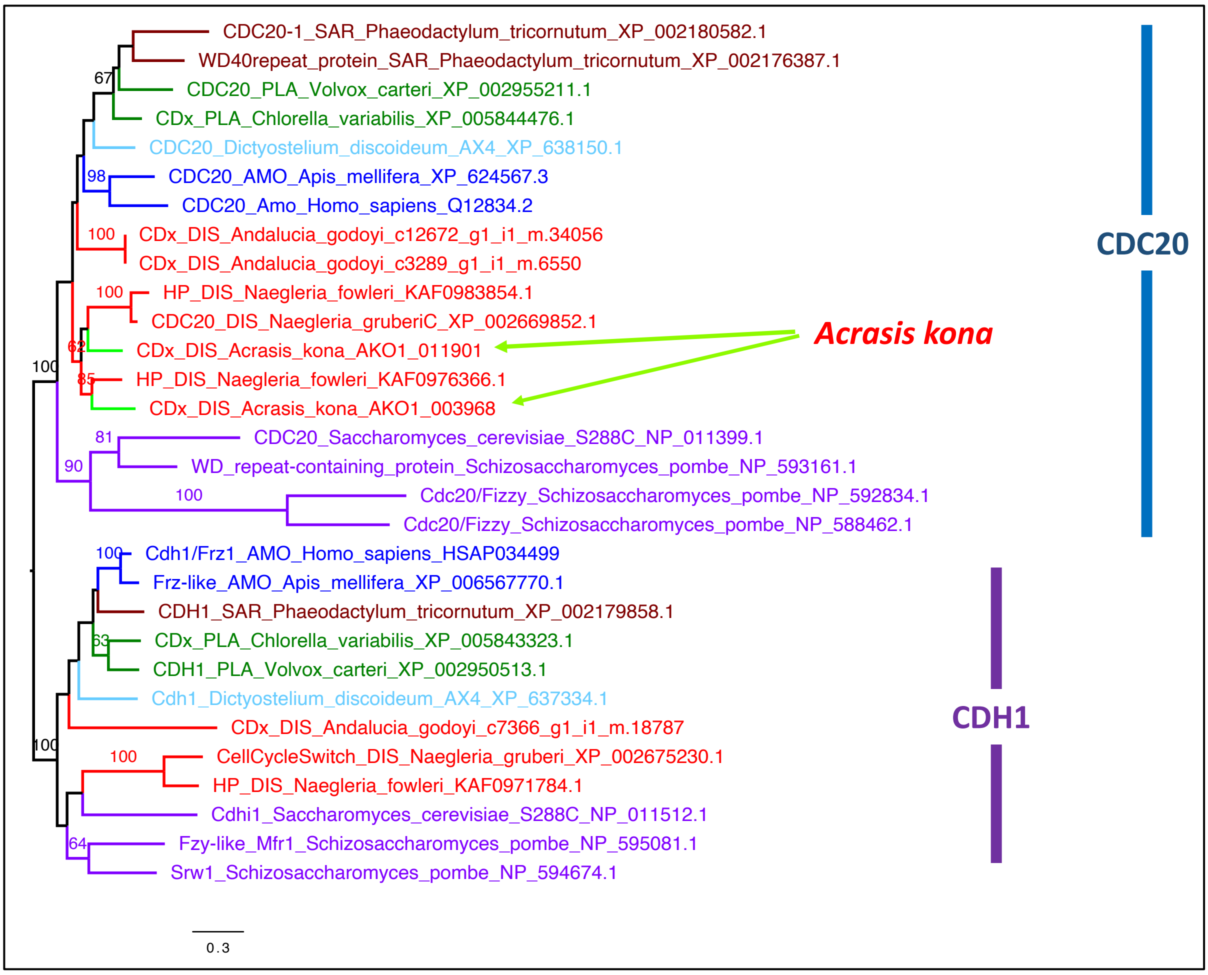

Figure S7. The central cell divison regulatory proteins CDC20 and CDH1 of *Acrasis kona* are both CDC20s. Sequences were identified by taxid-limited BLASTp with human protein sequence queries, and all hit sequences with e- values < e^{-35} were retrieved. The tree was constructed from 306 core aligned positions using RAxML and the LG+g model [104]. Sequences are labeled with their GenBank annotation (otherwise denoted as hypothetical proteins or HP), and colored according to major taxonomy as follows: Stramenopila, Alveolata and Rhizaria (SAR; brown), Chlorophyta (PLA, green), Discoba (DIS, red), Amorphea (Amo, aqua). Numbers on branches show bootstrap values over 60%. The tree is drawn to scale according to the scale bar at the bottom (substitutions per site).

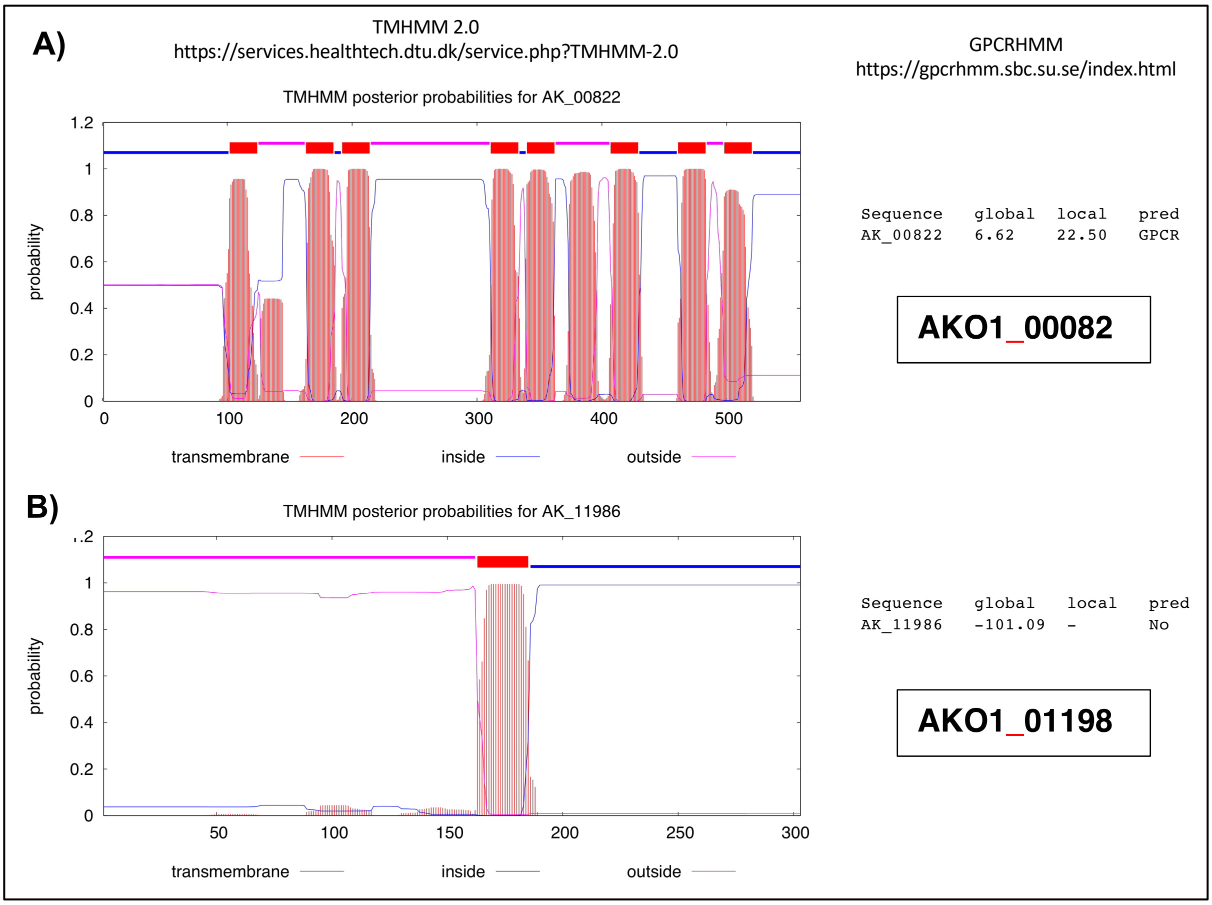

Figure S8. External signaling proteins predicted among novel *Acrasis kona* Aggup accessions. All unannotated *A. kona* aggregation proteins with predicted transmembrane domains (Supplementary Tables S13, S24) were analyzed using the Hidden Markov Model based GPCR predictor TMHMM 2.0 (services.healthtech.dtu.dk/service.php?TMHMM-2.0 (Wistrand et al. 2006). A) AKO1_000822 is strongly predicted to encode a classic GPCR with 8-9 transmembrane domains and large loops on both sides of the plasma membrane. B) AKO1_011986 is predicted to encode a membrane signaling or receptor protein with a single membrane spanning domain and large domains on both external and internal membrane surfaces.

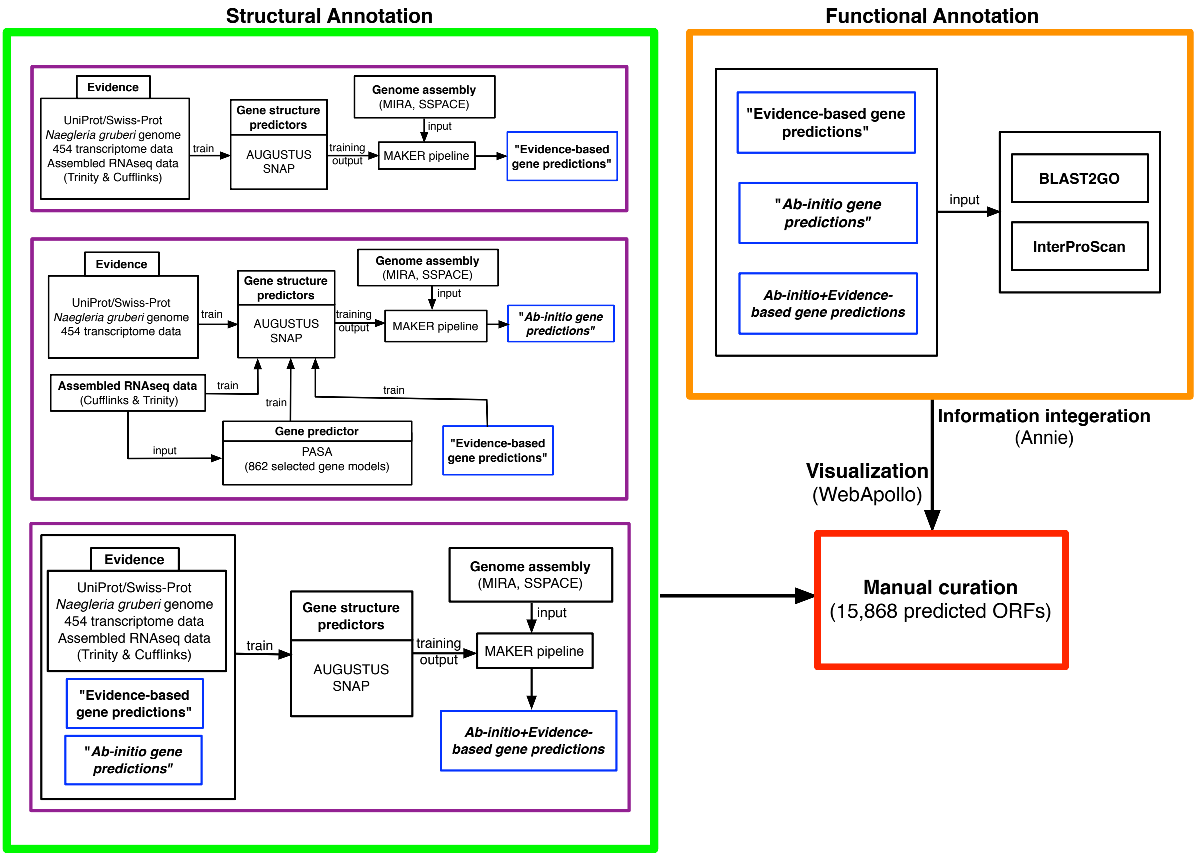

Figure S9. The *Acrasis kona* genome annotation pipeline. The pipeline shown was used to perform structural (left) and functional (right) annotation followed by manual curation of the *A. kona* nuclear genome. Three different strategies were used to train gene predictors for structural annotation to produce evidence-based gene predictions, *ab-initio* gene predictions and evidence+*ab-initio* gene predictions.
