# Supplementary material for: The Acrasis kona genome and developmental transcriptomes reveal deep origins of eukaryotic multicellular pathways
Source: Nat Commun. 2024 Nov 25;15:10197. doi: 10.1038/s41467-024-54029-z (PMC11589745; doi:10.1038/s41467-024-54029-z)
Supplement: Supplementary file 2 — Reporting Summary [file 41467_2024_54029_MOESM2_ESM.pdf]

nature portfolio

Corresponding author(s): Sandra L Baldauf

Last updated by author(s): Jul 3, 2024

Reporting Summary

Nature Portfolio wishes to improve the reproducibility of the work that we publish. This form provides structure for consistency and transparency in reporting. For further information on Nature Portfolio policies, see our Editorial Policies and the Editorial Policy Checklist.

Statistics

For all statistical analyses, confirm that the following items are present in the figure legend, table legend, main text, or Methods section.

n/a	Confirmed	
☐	☒	The exact sample size (*n*) for each experimental group/condition, given as a discrete number and unit of measurement
☐	☒	A statement on whether measurements were taken from distinct samples or whether the same sample was measured repeatedly
☒	☐	The statistical test(s) used AND whether they are one- or two-sided<br>*Only common tests should be described solely by name; describe more complex techniques in the Methods section.*
☒	☐	A description of all covariates tested
☒	☐	A description of any assumptions or corrections, such as tests of normality and adjustment for multiple comparisons
☒	☐	A full description of the statistical parameters including central tendency (e.g. means) or other basic estimates (e.g. regression coefficient) AND variation (e.g. standard deviation) or associated estimates of uncertainty (e.g. confidence intervals)
☒	☐	For null hypothesis testing, the test statistic (e.g. *F*, *t*, *r*) with confidence intervals, effect sizes, degrees of freedom and *P* value noted<br>*Give P values as exact values whenever suitable.*
☒	☐	For Bayesian analysis, information on the choice of priors and Markov chain Monte Carlo settings
☒	☐	For hierarchical and complex designs, identification of the appropriate level for tests and full reporting of outcomes
☒	☐	Estimates of effect sizes (e.g. Cohen's *d*, Pearson's *r*), indicating how they were calculated

Our web collection on statistics for biologists contains articles on many of the points above.

Software and code

Policy information about availability of computer code

Data collection	n/a
Data analysis	FASTX toolkit (v0.0.13) (hannonlab.cshl.edu/fastx_toolkit/). Trimmomatic (v0.32) (Bolger et al. 2014). MIRA (v3.9.9) (Chevreux et al. 2004). SSPACE (v2.0) (Boetzer et al. 2011). QUAST (Gurevich et al. 2013). Trinity (version 2014-07-17). FastQC (v0.11.8) (Andrews 2014), STAR (version 2.5.2) (Dobin et al. 2013). featureCounts program in R (Liao et al. 2014). GFOLD (v1.1.4) (Feng et al. 2012). RepeatRunner (Smith et al. 2007). RepeatMasker (www.repeatmasker.org/). Augustus (version 2.7) (Stanke and Morgenstern 2005). SNAP (November 2013 release) (Korf 2004). Maker pipeline (Cantarel et al. 2008). PASA package (Haas et al. 2003). Web Apollo (Lee et al. 2013). EMBOSS stretcher (Rice et al. 2000). Tablet (Milne et al. 2013). NBIS functional annotation pipeline (https://github.com/NBISweden/pipelines-nextflow). BLASTp at NCBI (https://www.ncbi.nlm.nih.gov/). InterProScan (version 5.7-48). Gene Ontology (GO) (Ashburner et al. 2000). Blast2GO (Conesa et al. 2005). Annotation Information Extractor (Annie) (Ooi et al. 2009). BlastKOALA (Kanehisa et al. 2016). iPath (Letunic et al. 2008). OrthoMCL (Li et al. 2003). ProteinOrtho (Lechner et al. 2011). SignalP (version 4.0) (Petersen et al. 2011). Phobius webserver (Käll et al. 2007). GoFeat (Araujo et al. 2018). MUSCLE (v3.8.32) (Edgar 2004). AliView (Larsson 2014). trimAL (Capella-Gutiérrez et al. 2009). IQTree (Chernomor et al. 2016). RAxML(Stamatakis 2006). SICLE (DeBlasio and Wisecaver 2016). GhostKOALA (https://www.expasy.org/). TMHMM 2.0 (Krogh et al. 2001). GPCRHMM (Wistrand et al. 2006).

For manuscripts utilizing custom algorithms or software that are central to the research but not yet described in published literature, software must be made available to editors and reviewers. We strongly encourage code deposition in a community repository (e.g. GitHub). See the Nature Portfolio guidelines for submitting code & software for further information.

Data

Policy information about availability of data

All manuscripts must include a data availability statement. This statement should provide the following information, where applicable:

- Accession codes, unique identifiers, or web links for publicly available datasets
- A description of any restrictions on data availability
- For clinical datasets or third party data, please ensure that the statement adheres to our policy

The Acrasis kona whole genome shotgun project has been deposited at DDBJ/ENA/GenBank under the accession JAOPGA000000000. The version described in this paper is version JAOPGA020000000. Transcriptome data for this paper are deposited in SRA files SRR22861965, SRX19285604, SRX19285605, and SRX19285603. Acrasis kona strain MYA-3509 (formerly Acrasis rosea) is available from the American Type Culture Collection (ATCC:MYA-3509).

Research involving human participants, their data, or biological material

Policy information about studies with human participants or human data. See also policy information about sex, gender (identity/presentation), and sexual orientation and race, ethnicity and racism.

Reporting on sex and gender	n/a
Reporting on race, ethnicity, or other socially relevant groupings	n/a
Population characteristics	n/a
Recruitment	n/a
Ethics oversight	n/a

Note that full information on the approval of the study protocol must also be provided in the manuscript.

Field-specific reporting

Please select the one below that is the best fit for your research. If you are not sure, read the appropriate sections before making your selection.

☒ Life sciences ☐ Behavioural & social sciences ☐ Ecological, evolutionary & environmental sciences

For a reference copy of the document with all sections, see nature.com/documents/nr-reporting-summary-flat.pdf

Life sciences study design

All studies must disclose on these points even when the disclosure is negative.

Sample size	A single isolate of a single species was sequenced.
Data exclusions	no data were excluded
Replication	Sequencing used independent runs of 454 GS Titanium and Illuminna. Developmental RNAseq used single replicates of three developmental stages. Differential expression analysis used G-fold to compensate for single replicates.
Randomization	n/a
Blinding	n/a

Reporting for specific materials, systems and methods

We require information from authors about some types of materials, experimental systems and methods used in many studies. Here, indicate whether each material, system or method listed is relevant to your study. If you are not sure if a list item applies to your research, read the appropriate section before selecting a response.

Materials & experimental systems

n/a	Involved in the study
☒	☐ Antibodies
☒	☐ Eukaryotic cell lines
☒	☐ Palaeontology and archaeology
☐	☒ Animals and other organisms
☒	☐ Clinical data
☒	☐ Dual use research of concern
☒	☐ Plants

Methods

n/a	Involved in the study
☒	☐ ChIP-seq
☒	☐ Flow cytometry
☒	☐ MRI-based neuroimaging

Animals and other research organisms

Policy information about studies involving animals; ARRIVE guidelines recommended for reporting animal research, and Sex and Gender in Research

Laboratory animals	no animals were used in this study
Wild animals	n/a
Reporting on sex	n/a
Field-collected samples	n/a
Ethics oversight	n/a

Note that full information on the approval of the study protocol must also be provided in the manuscript.

Plants

Seed stocks	no plants were used in this study
Novel plant genotypes	n/a
Authentication	n/a
