# Supplementary material for: The Acrasis kona genome and developmental transcriptomes reveal deep origins of eukaryotic multicellular pathways
Source: Nat Commun. 2024 Nov 25;15:10197. doi: 10.1038/s41467-024-54029-z (PMC11589745; doi:10.1038/s41467-024-54029-z)
Supplement: Supplementary file 3 — Description of Additional Supplementary Files [file 41467_2024_54029_MOESM3_ESM.pdf]

## Description of Additional Supplementary Files

### Supplementary Data 1.

Description: Predicted membrane proteins in Acrasis kona and Naegleria gruberi. The number of transmembrane domains (TM) and presence/absence (Y/0) of signal peptide sequences (SP) was predicted for the full proteomes of A. kona and N. gruberi using Phobius (Käll et al. 2007). A. kona proteins assigned to multiprotein families (Orhology Groups, Supplemental Data D2) are indicated by the orthology group number (ID).

### Supplementary Data 2.

Description: Acrasis kona orthologous protein clusters and their presence/absence and size in other eukaryotes. Orthologous groups (OG) were identified in A. kona and diverse eukaryotes (Table S5) by protein clustering using ProteinOrtho (Lechner et al. 2011). Columns C-V summarize numbers of sequences for each taxon. Key to taxon labels is shown to the right of the table (columns x-y).

### Supplementary Data 3.

Description: Full set of predicted Acrasis kona metabolic pathways (BLASTKoala). Expasy enzyme numbers were assigned to all non-novel A. kona proteins using BLASTKoala (www.kegg.jp/blastkoala/, Kanehesa et al. 2016). The Expasy numbers were then input to IPATH (pathways.embl.de/) to generate the metabolic map shown in Figure S1.

### Supplementary Data 4.

Description: Numbers of signalling domains in Acrasis kona compared to Naegleria gruberi and diverse other eukaryotes (Figure 4). Signalling domains were identified in all genomes using InterProScan with default settings. The number of domains with an IPR ID in column S were calculated using in-house scripts.

### Supplementary Data 5.

Description: Acrasis kona gene expression levels for three life cycle stages and differential expression between them. Differential expression levels were calculated using GFOLD (v1.1.4).

**Supplementary Data 6.**

Description: Acrasis kona accessions with substantial differential expression (SDE). SDE between life cycle stages (Gro to Agg and Agg to Germ) was defined using a combination of RPKM and differential expression values (DE) calculated using GFOLD (v1.1.4). SDE criteria were as follows: RPKM>10,000 and DE_Log2 >0.1, RPKM 1000-9999 and DE_Log2 ≥0.25; RPKM 50-999 and DE_Log2 ≥ 0.9; RPKM 10-49 and DE_Log2 ≥1.45; RPKM 2.0-9.99; DE_Log2 ≥ 2.0 and Reads >100).

**Supplementary Data 7.**

Description: Annotation of Acrasis kona aggregation accessions (SDE Aggup, Figure 5). Accessions with substantially increased expression from growth to aggregation (SDE Aggup) and/or very highly expressed during aggregation (Supplemental Data D6) were annotated based on a consensus of their top BLASTp hits to GenBank nr, human Swissprot and Dictyostelium discoideum AX4 (Ddi) RefSeq databases, plus DictyBase annotation of Ddi top hits. Non-SDE accession important for developmental signalling are included as additional information only and are shown in italics. Horizontal transfers confirmed by phylogeny are indicated by their bootstrap support. Further abbreviations are as follows: TM (transmembrane domain, Data D1), SP (signal peptide, Data D1), URF (unidentified reading frame), OG (orthology group, Data D2), RNAseq (Data D5), OG:Ako (OG size in Acrasis kona), OG:Ngr (OG size in Naegleria gruberi), OG:other euks (OG size across diverse eukaryotes, Data D2), cvg Q:S (BLASTp coverage for query and subject).

**Supplementary Data 8.**

Description: Phylogenetic trees supporting horizontal transmission of Acrasis kona Aggup sequences. Homologous sequences were acquired by BLASTp against the GenBank nr or nrcluster database, in some cases using taxid constraints. Amino acid sequences were aligned using MUSCLE, trimmed using TrimA1 with default settings and used to derive trees using the LG+gamma substitution model and RAxML (www.phylo.org/) or IQTree (iqtree.cibiv.univie.ac.at/). Trees are shown as nexus formatted files, and in some cases also as figures.

**Supplementary Data 9.**

Description: Predicted eukaryotic autophagosome, associated proteins and their taxonomic distribution and conservation across eukaryotes (Figure 6). Initial BLASTp queries were selected using GenBank annotation in four model organisms - Dictyostelium discoideum AX4 (Ddi), Saccharomyces cerevisiae S288C (Sce), Schizossacharomyces pombe (Spo) and human (Hsa) and BLASTed against each other. Based on strong sequence similarity between Ddi and Hsa sequences and

smaller numbers of paralogs in Ddi, Ddi sequences were selected for the final set of queries. Ddi queries were searched against a wide taxonomic diversity of eukaryotes using species or genus level taxid limits. Hits were scored for e-value and percent coverage of query and subject (cvg Q:S). Sequences not retrieved by BLASTp but annotated as the same protein are taken from Fischer and Eichinger (2019) and Cheng et al (2017) and denoted “annotation”. Gene expression levels for A. kona are from Supplemental Data file 4 and for Ddi from Santhanam et al. (2015).

**Supplementary Data 10.**

Description: The Acrasis kona predicted proteasome: differential expression and sequence conservation relative other eukaryotes (Figure 7). The presence and sequence conservation of proteasomal proteins was surveyed across eukaryotes using BLASTp with an e-value cut-off of e-5. All BLASTp searches used human Refseq queries except where there was no human homolog, in which case Saccharomyces cerevisiae (Sce) queries were used (denoted by *). Hits are scored for e-value and query coverage (cvg). Identity of alpha and beta is based on top hit only. The table is adapted and expanded from Bard et al. (2018), except for taxon specific genes which are excluded (Sce 20S assembly proteins Fub1p/PI31, POC1, POC2, POC4, Irc25p, Ump1, and Hsa 20S assembly protein PAC3).

**Supplementary Data 11.**

Description: The Acrasis kona exosome: differential expression and sequence conservation relative to other eukaryotes (Figure S5). The distribution and conservation of exosomal proteins was surveyed across eukaryotes using human (Hsa) RefSeq queries except RdRP, which lacks a human homolog. BLASTp hits are scored for e-value and query coverage (cvg). Sequences that lack a significant BLASTp hit but are nonetheless annotated as the same protein are denoted as "annotated". Structure is taken from Budenholzer et al. (2017). Gene expression levels for A. kona are from Supplemental Data file D4 and for Ddi from Santhanam et al. (2015).

**Supplementary Data 12.**

Description: Acrasis kona Aggup cell cycle accessions. Aggup accessions annotated with potential cell cycle function are taken from Supplemental Data file D7 along with differential expression (DE) and length corrected read (RPKM) values. Dddi AX4 DE and RPKM values were derived from Santhanam et al. (2015).

**Supplementary Data 13.**

Description: The Acrasis kona kinetochore, differential expression and sequence conservation relative to other eukaryotes (Figure S6). The distribution and conservation of kinetochore component proteins was surveyed across eukaryotes using BLASTp with human (Hsa) RefSeq queries. Queries were retrieved from the the database of van Hooff et al. (2017). BLASTp hits are scored for e-value and query coverage (cvg). A. kona DE and RPKM are from Supplemental data file 4. D. discoideum DE and RPKM values were derived from Santhanam et al. (2015).

**Supplementary Data 14.**

Description: Aggregation signalling in Acrasis kona. Data are extracted from Table S11 and graphically summarized in Figure 7. Accessions shown in italics and grey font are not strongly Aggup but substantially expressed during aggregation with central roles in several signalling pathways.

**Supplementary Data 15.**

Description: Acrasis kona Aggup proteins potentially involved in construction of the extracellular matrix. Annotation is extracted from Supplementary Data file D7.

**Supplementary Data 16.**

Description: Novel aggregation-specific accessions. Aggregation-specific is defined as accessions with both Aggup >1.0 and Germdn < -1.0. Pairs of accessions belonging to the same orthology group (OG) are highlighted in gold.

**Supplementary Data 17.**

Description: Stage-specific expression of multi-copy Acrasis kona accessions. All Aggup accessions belonging to orthology groups (OGs) are shown. Information includes differential expression values (DE) for growth versus aggregation (Gro v Agg) and aggregation versus germination (Agg v Germ), number of length corrected reads (RPKM), orthology group designation (OG#), and number of accessions in the OG for Acrasis kona, Naegleria gruberi, and other examined eukaryotes plus the range of OG size among them (Table S5). OGs with multiple or all members Aggup are indicated by blue and gold background, respectively.

**Supplementary Data 18**

Description: Full Acrasis kona proteome in fasta format.

**Supplementary Movie 1**

Description: A real time video of Acrasis kona co-cultured with Dictyostelium discoideum
